# A Hierarchical FSM–BT Control Architecture for a Passive/Active Rehabilitation Exoskeleton

**DOI:** 10.3390/s26185731

**Published:** 2026-09-09

**Authors:** Regina B. M. Chávez-Reynoso, Dennis Barrios-Aranibar, Raquel E. Patiño-Escarcina

**Affiliations:** Electrical and Electronic Engineering Department, Universidad Católica San Pablo (UCSP), Arequipa 04001, Peru; dbarrios@ucsp.edu.pe (D.B.-A.); rpatino@ucsp.edu.pe (R.E.P.-E.)

**Keywords:** rehabilitation robotics, behavior trees, finite-state machines, hybrid control architecture, hierarchical control, Software-in-the-Loop, exoskeleton control

## Abstract

Rehabilitation exoskeletons require supervisory control that can preserve globally safe operating modes while executing local therapeutic behaviors reactively. Pure Finite-State Machine (FSM) implementations provide explicit mode supervision but become increasingly coupled as behaviors and recovery transitions are added, whereas Behavior Trees (BTs) provide modular reactive execution but do not inherently encode a unique global operating mode. This work presents an application-specific hierarchical FSM–BT architecture for the SARA passive–active lower-limb rehabilitation platform. The novelty is not the generic combination of FSMs and BTs; rather, it lies in the rehabilitation-oriented separation of global therapeutic-mode supervision and event-priority safety arbitration from parameterizable BT execution and local recovery before escalation. The architecture was evaluated exclusively in a custom Python Software-in-the-Loop (SIL) environment using an eight-degree-of-freedom second-order joint model, a 100 Hz plant integration rate, a 10 Hz supervisory FSM, and 50 Hz BT execution. Thirty Monte Carlo trials were performed for each of four scenarios and for three functionally equivalent architectures (FSM-only, BT-only, and FSM–BT), for 360 comparative trials with a fixed random seed. The proposed FSM–BT architecture achieved a 98.3% overall trial-success rate, compared with 89.2% for FSM-only and 92.5% for BT-only. Its mean software-level unsafe-condition-to- Emergency Stop response was 71.9 ms, significantly lower than the 159.7 ms FSM-only response ($p<10^{-8}$, paired Wilcoxon test), while BT-only produced the same safety timing because it used the same 50 Hz local evaluation cadence. The active-assistance model yielded a mean eight-DOF angular RMSE of 2.42°. A structural extension study further required 54 edit operations to add six therapeutic routines to FSM–BT, versus 60 for BT-only, 96 for a hierarchical state-machine baseline, and 138 for FSM-only.

## 1. Introduction

Lower-limb rehabilitation exoskeletons can provide repetitive and customizable movement training; therefore, their control architecture must coordinate multiple modes of operation, sensing, therapeutic actions, and safety constraints, all while interacting with the user [1,2,3]. The monitoring problem becomes more difficult as different exercises, recovery behaviors, and conditions for monitoring the user are incorporated, since the control policy must remain interpretable and must prevent local behaviors from deviating from the currently authorized therapeutic mode.

Finite-State Machines (FSMs) are widely used in rehabilitation robotics because their precise states and conditional transitions allow for deterministic management of phases and modes [4,5]. Their principal limitation is structural coupling: when local exceptions, recovery paths, and new behaviors are represented directly as additional states and transitions, the policy can become difficult to extend and maintain. Behavior Trees (BTs) provide an alternative hierarchical representation based on reusable control, condition, and action nodes that are reevaluated cyclically [6,7,8]. BTs therefore offer strong modularity and reactivity, but a monolithic BT does not inherently provide the same explicit representation of mutually exclusive global therapeutic modes or prioritized global safety transitions.

Hybrid FSM–BT patterns have already been studied in other robotic domains; consequently, this manuscript does not claim novelty for the generic idea of combining an FSM with BTs [9,10,11]. The specific research question addressed here is whether a rehabilitation-oriented supervisor–executor decomposition can provide measurable advantages when the same SARA task set is encoded as FSM-only, BT-only, or hierarchical FSM–BT policies. The target SARA architecture contains passive and active therapeutic modes, motion execution, local recovery, and safety escalation. The proposed FSM retains explicit authority over global-mode changes and critical events, whereas each authorized state activates a parameterizable BT that performs local sensing checks, motion-reference execution, monitoring, and bounded recovery.

The main contributions of this work are as follows:

1. A rehabilitation-specific hierarchical mapping in which an FSM represents global therapeutic modes, safety-priority events, and escalation states, while BTs encapsulate local therapeutic execution and bounded recovery;
2. Explicit BT node-design and extension rules that distinguish condition, action, control, and recovery nodes and define when new functionality belongs to the global FSM or to a local BT;
3. A mathematical and algorithmic description of the FSM, BT, hybrid supervisor–executor interaction, low-level joint model, and software-level safety response;
4. A reproducible Python Software-in-the-Loop (SIL) validation with defined dynamics, sensing noise, communication uncertainty, update periods, failure injection, success criteria, confidence intervals, and statistical tests;
5. An ablation and scalability comparison against functionally equivalent FSM-only and BT-only policies, supplemented by a structural hierarchical state-machine (HSM) baseline.

The validation reported in this paper is entirely software-based. Neither the STM32H753ZI nor the ZED Box executed the controller, and no timing value was measured from a physical actuator or communication bus. No human participant was involved. The hardware architecture shown later is included because it defines the intended SARA deployment interfaces and sensing arrangement; it should not be read as evidence of hardware execution. The distinction between the intended platform and the SIL implementation is kept throughout the manuscript.

The present paper is structured into six sections, organized as follows: Section 1 introduces the research topic, providing a general overview and presenting the research problem. Section 2 reviews current control architectures for rehabilitation robotics, with particular emphasis on Finite-State Machines (FSMs), Behavior Trees (BTs), and hybrid approaches combining these methods. Section 3 presents the proposed hierarchical FSM-BT architecture, including its mathematical formulation and the interaction between global supervision and local behaviors.

Section 4 describes the implementation of the proposed architecture to the SARA robotic system, including the simulation model, sensor configuration, safety mechanisms, and Software-in-the-Loop (SIL) implementation. Section 5 presents the simulation methodology, the comparative evaluation against architectures based solely on FSMs and BTs, the scalability assessment, and a discussion of the results obtained. Finally, Section 6 summarizes the main conclusions, limitations, and directions for future work.

## 2. State of the Art

This section focuses on the roles and limitations of Finite-State Machines (FSMs) and Behavior Trees (BTs) that are directly relevant to rehabilitation robotics, rather than providing a general tutorial. FSMs remain attractive for deterministic global supervision, whereas BTs provide modular and reactive local execution. The central design question for a rehabilitation exoskeleton is therefore how to preserve explicit operating-mode and safety semantics while avoiding the structural growth and maintenance burden of encoding all therapeutic behavior as a single state-transition graph [6,11,12]. Hierarchical robotic architectures naturally separate these supervisory decisions from lower-level trajectory and actuator control, which motivates the two-level organization evaluated in this work.

### 2.1. Finite-State Machines

FSMs describe behavior through explicit states and guarded transitions, making operating modes, sequencing, and safety escalation interpretable and deterministic [5,13]. Table 1 shows why this structure remains useful in locomotion, mission planning, human–robot interaction, and safety supervision. Its principal limitation for the present application is structural growth: when local therapeutic exceptions, retries, sensing checks, and recovery branches are represented as additional states and transitions, the supervisory graph becomes increasingly coupled. Hierarchical FSMs reduce but do not eliminate this maintenance problem. Consequently, this work retains the FSM only for global therapeutic context and safety-critical mode transitions rather than using it to encode every local action.

### 2.2. Behavior Trees

BTs organize control-flow nodes and condition/action leaves in a hierarchical tree that is reevaluated by periodic ticks, supporting modular reuse and rapid local reaction [6,14]. Table 2 summarizes representative robotic uses. Their modularity is advantageous when rehabilitation behaviors must be extended with new checks, actions, or bounded recovery routines; however, the behavior still depends on correct tree design, and a BT-only controller does not inherently provide the explicit global operating-mode semantics desired for safety-critical therapy supervision. This motivates assigning local execution to BTs while reserving global-mode arbitration for the FSM.

The studies in Table 2 reinforce this complementary role: BTs are effective for strategy/task execution that benefits from modular conditions and reusable actions, whereas global supervision and safety escalation remain clearer when represented explicitly. For rehabilitation robotics, this separation is particularly relevant because local posture, interaction, and sensing conditions may change during a therapy, while the permissible operating mode must remain unambiguous.

**Table 1:** Use of Finite-State Machines in robotics.

Application	Level Within the Robotic Architecture	Technical Advantages	Technical Disadvantages	Technical Observations	References
Robotic locomotion control	**Trajectory planning.** At this level, the FSM organizes the discrete sequence of motion phases (stance, swing, transition).	Enables clear synchronization between mechanical events and controllers, ensuring temporal coherence in periodic motions.	The number of states increases rapidly when external disturbances or environmental variations are considered.	Each state usually executes a specific position, velocity, or torque controller.	[4,5]
Robotic mission planning	**Strategy planning.** The FSM defines the global logical sequence of tasks that the robot must execute throughout a complete mission.	Deterministic and formally verifiable behavior.	Low adaptability to unforeseen events.	Frequently combined with temporal logic and formal verification.	[9,15]
Autonomous mobile robots	**Path planning.** The FSM governs transitions between navigation behaviors such as exploration, path following, and obstacle avoidance.	Clear structure for discrete decision-making.	Difficulty in scaling to complex behaviors.	Hierarchical FSMs reduce design complexity.	[12,16]
Human–robot interaction	**Strategy planning.** The FSM models interaction states defined by discrete events originating from the user or the environment.	Predictable and easily interpretable behavior.	Limited handling of uncertainty and human ambiguity.	Suitable for well-structured interaction protocols.	[17,18]
Safety supervision	Used as a supervisory layer that monitors critical conditions and forces transitions to safe states.	Deterministic response to faults.	Rigid behavior in unforeseen contexts.	Used as a higher-level layer over BTs or reactive controllers.	[19,20]

**Table 2:** Use of Behavior Trees in robotics.

Application	Level Within the Robotic Architecture	Technical Advantages	Technical Disadvantages	Technical Observations	References
Reactive behavior control	**Strategy planning.** The BT coordinates high-level decisions through continuous node evaluation, dynamically reacting to the environment.	High reactivity and modularity.	Limited formal verification.	The tick mechanism enables constant reevaluation.	[7,14]
Service robotics	**Strategy planning.** BTs organize complex and long-duration tasks through hierarchies of reusable behaviors.	High scalability and reusability.	Complex debugging in large trees.	Widely used in service platforms.	[8,21]
Robotic manipulation	**Task planning.** The BT coordinates discrete action sequences while encapsulating continuous control within leaf nodes.	Natural discrete–continuous integration.	Strong dependence on tree design.	Actions often execute local planning.	[22,23]
Complex autonomous robots	**Strategy planning.** The BT manages multiple perceptual and control subsystems.	Robustness in dynamic environments.	Expert-dependent design.	Frequently combined with symbolic planning.	[24,25]

### 2.3. Comparative Studies Between FSMs and BTs

Comparative studies distinguish between FSMs and BTs based on their structural properties, rather than assuming that one representation predominates across all tasks. FSMs provide explicit semantics of the global state and deterministic transitions with conditions, while BTs offer a hierarchical interface that facilitates local modification, the reuse of subtrees, and rapid reevaluation of conditions [8,9,11]. Iovino et al. quantitatively examined programming effort and structural modification when equivalent robot tasks were represented using FSMs and BTs, showing that BT modularity can reduce the number of policy edits required as tasks are extended [11]. Hallen et al. further reported that combining BT execution with FSM-style high-level mode organization can improve readability in a complex industrial robotic application [10].

These results motivate, but do not by themselves prove, the suitability of a hybrid architecture for rehabilitation. Rehabilitation exoskeletons add requirements that are less central in many autonomous-robot benchmarks: mutually exclusive therapeutic modes, direct physical human–robot interaction, bounded local recovery, explicit emergency escalation, and parameterized motion execution. The gap addressed by the present study is therefore not the invention of an FSM–BT pattern but, rather, its rehabilitation-specific organization and a controlled comparison of equivalent FSM-only, BT-only, and FSM–BT encodings under the same SIL disturbances.

Table 1 and Table 2 summarize the strengths and weaknesses that motivate the role assigned to each formalism. The closest hybrid works are summarized in Table 3. A quantitative comparison based on trial success, software safety-response time, recovery success, global-mode consistency, and structural edit cost is presented in Section 5; the comparison therefore relies on defined numerical metrics rather than arbitrary qualitative scores.

### 2.4. Behavior and Action Models in Robotic Exoskeletons

Rehabilitation exoskeletons combine direct physical human–robot interaction with multiple sensing, actuation, and therapeutic modes, making supervisory organization a safety-relevant design problem. Table 4 summarizes the main behavior representations encountered in this domain. FSMs remain common for gait phases and operating modes because their discrete transitions are interpretable and straightforward to guard [5,29]. Hierarchical state machines reduce part of the resulting state proliferation, but local exceptions and recovery paths can still enlarge the transition structure as the therapy becomes more complex.

BTs provide a complementary representation for local task execution because conditions, actions, and recovery branches can be encapsulated as reusable subtrees and reevaluated at each tick [7]. Adaptive methods such as rule-based assistance, probabilistic inference, and reinforcement learning can operate within these structured supervisors rather than replacing the safety-oriented supervisory layer [2,18,32,33]. This distinction is important because recent knee-exoskeleton studies show that low-level disturbance rejection and motion tracking already require dedicated control design [34,35]; the present study addresses the complementary problem of how therapeutic modes, local behaviors, recovery, and safety escalation are organized above that low-level controller.

The motivation derived from Table 1, Table 2, Table 3 and Table 4 is therefore specific: an FSM is retained where explicit global therapeutic context is valuable, while BTs are used where local extension and reactive recovery would otherwise increase supervisory coupling. The proposed method does not replace adaptive joint control or claim that the generic FSM–BT pattern is novel; it evaluates this rehabilitation-specific allocation quantitatively in Section 5.

## 3. Proposal

The analysis of the state of the art indicates that rehabilitation-oriented robotic systems require control architectures capable of managing sequential operating phases while also responding reactively to changes in the user, the task, and the environment. In this context, the exclusive use of Finite-State Machines or Behavior Trees presents limitations when applied to complex human–robot interaction scenarios. FSMs provide deterministic supervision and clear state transitions, but their structure may become rigid and difficult to scale as the number of operating modes and safety conditions increases. Conversely, BTs offer modular and reactive execution, but they do not inherently provide a global supervisory structure for coordinating high-level operating modes.

### 3.1. Mathematical Formulation of the FSM–BT Hierarchy

The supervisory FSM is defined as

(1) $$M_{FSM}=\left(S,E,G,\delta ,s_{0}\right),$$

where *S* is the finite set of global operating states, *E* is the set of external and internal events, *G* is the set of transition guards, δ:S×E×G→S is the guarded transition function, and $s_{0}$ is the initial state. At the *k*th FSM update, with period $T_{FSM}$, the supervisor evolves according to

(2) $$s_{k+1}=\delta \;\left(s_{k},e_{k},g_{k}\right).$$

An event that is intentionally rejected because its guard is false is recorded as a *rejected request*, not as a controller failure.

A Behavior Tree associated with state *s* is represented as a rooted ordered tree $B_{s}=\left(V_{s},E_{s},r_{s}\right)$ whose nodes return

(3) $$\sigma_{v}\left(t_{j}\right)\in \left\{\mathrm{Success},\mathrm{Failure},\mathrm{Running}\right\},$$

at BT tick $t_{j}=jT_{BT}$. For a sequence node with children $c_{1},\ldots ,c_{m}$, evaluation proceeds from left to right until a child returns *Failure* or *Running*; the sequence returns *Success* only if all children succeed. A selector returns *Success* or *Running* as soon as the first child returns that status and returns *Failure* only if all children fail. These return-status semantics define the local reactive policy independently of the global FSM state.

The hybrid mapping is defined by

(4) $$\Gamma :S_{op}\to \left\{B_{1},\ldots ,B_{n}\right\},$$

where $S_{op}\subseteq S$ contains the operational states that activate BT modules. The active tree is therefore B(t)=Γ(s(t)). Local BT recovery is bounded by a maximum number of retries $R_{BT}$. If the same condition remains unresolved after $R_{BT}$ attempts, or if a critical safety guard becomes true, the BT emits an escalation event $e_{esc}$ that is consumed by the FSM. In the SIL implementation, $T_{FSM}=100$ ms and $T_{BT}=20$ ms; hence,

(5) $$T_{FSM}=5T_{BT},$$

which makes the previously qualitative relation $T_{FSM}\gg T_{BT}$ explicit.

### 3.2. Algorithmic Execution

Based on these considerations, this work proposes a hierarchical control architecture that combines FSMs and BTs within a structured two-level control scheme. The main contribution of the proposal lies in the separation of global supervision, local behavior execution, and safety-oriented decision-making. The FSM defines the operational context of the system, manages transitions between rehabilitation modes, and coordinates safety-related events. In contrast, the BTs execute the detailed behaviors associated with each state, continuously evaluating sensory conditions and adapting local actions to the current interaction scenario. Table 5 summarizes the main execution steps of each approach, including state management, behavior execution, safety monitoring, and recovery mechanisms.

**Figure 1:**
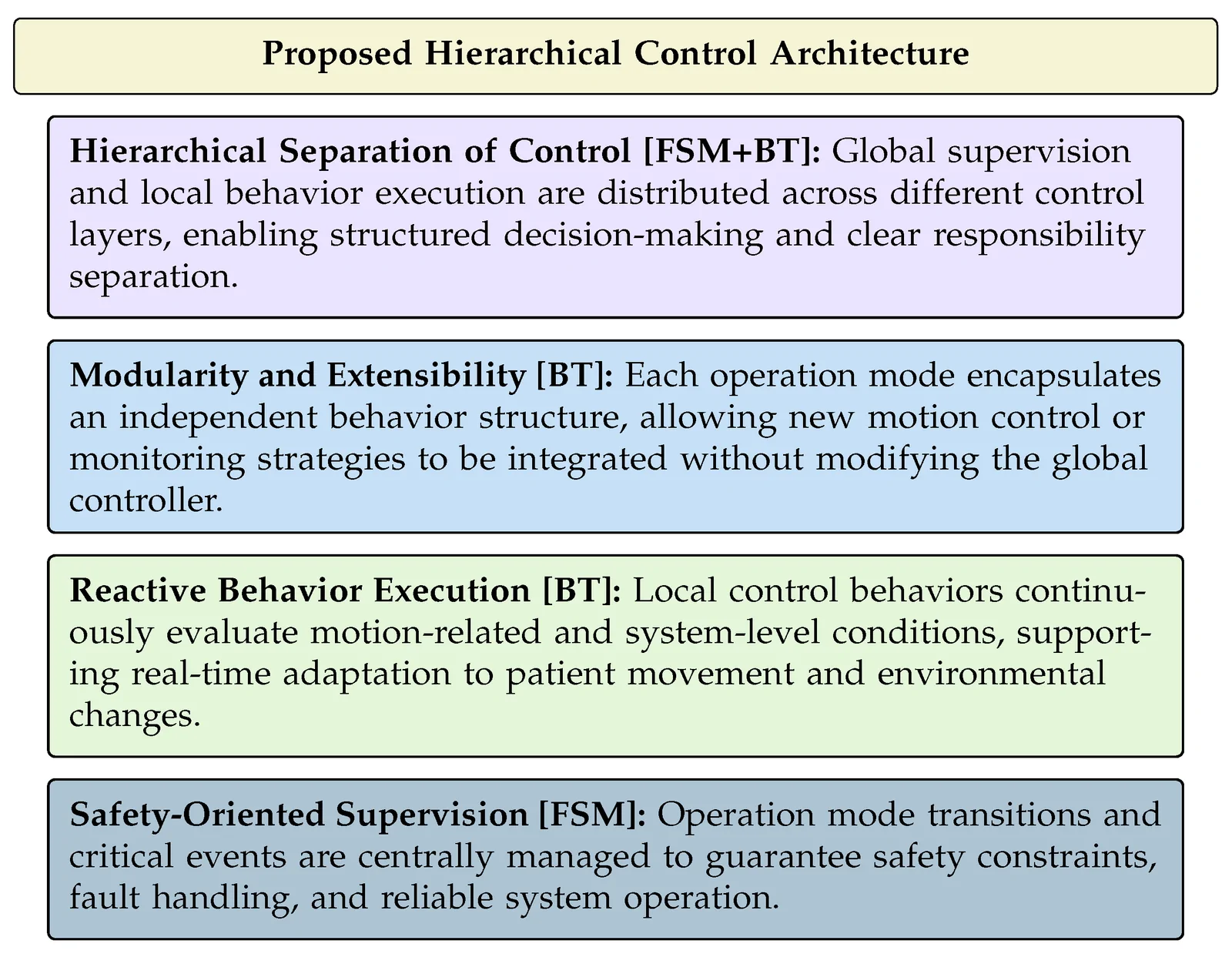
Design principles guiding the proposed hierarchical control architecture for rehabilitation exoskeleton.

The proposed architecture is designed according to four fundamental principles: hierarchical separation of control, modularity and extensibility, reactive behavior execution, and safety-oriented supervision. These principles are summarized in Figure 1, which establishes the conceptual basis for the proposed FSM–BT control strategy.

### 3.3. General Description of the Hierarchical Architecture

The proposed architecture is organized into two easily distinguishable control levels: a global supervision level and a local execution level. The global level is implemented using an FSM; its primary function is to monitor the system’s overall operational status. This FSM defines the main modes of operation and the transitions between states based on the requirements of each task, safety conditions, the therapist’s commands, and critical system events. The local level is associated with the states defined by the FSM. Rather than representing each state as a simple action, each state encapsulates a BT that remains active while the system operates within that specific state. These BTs handle the execution logic, which includes sensory evaluation, decision-making, the activation of local control actions, and the response to transient events.

**Figure 2:**
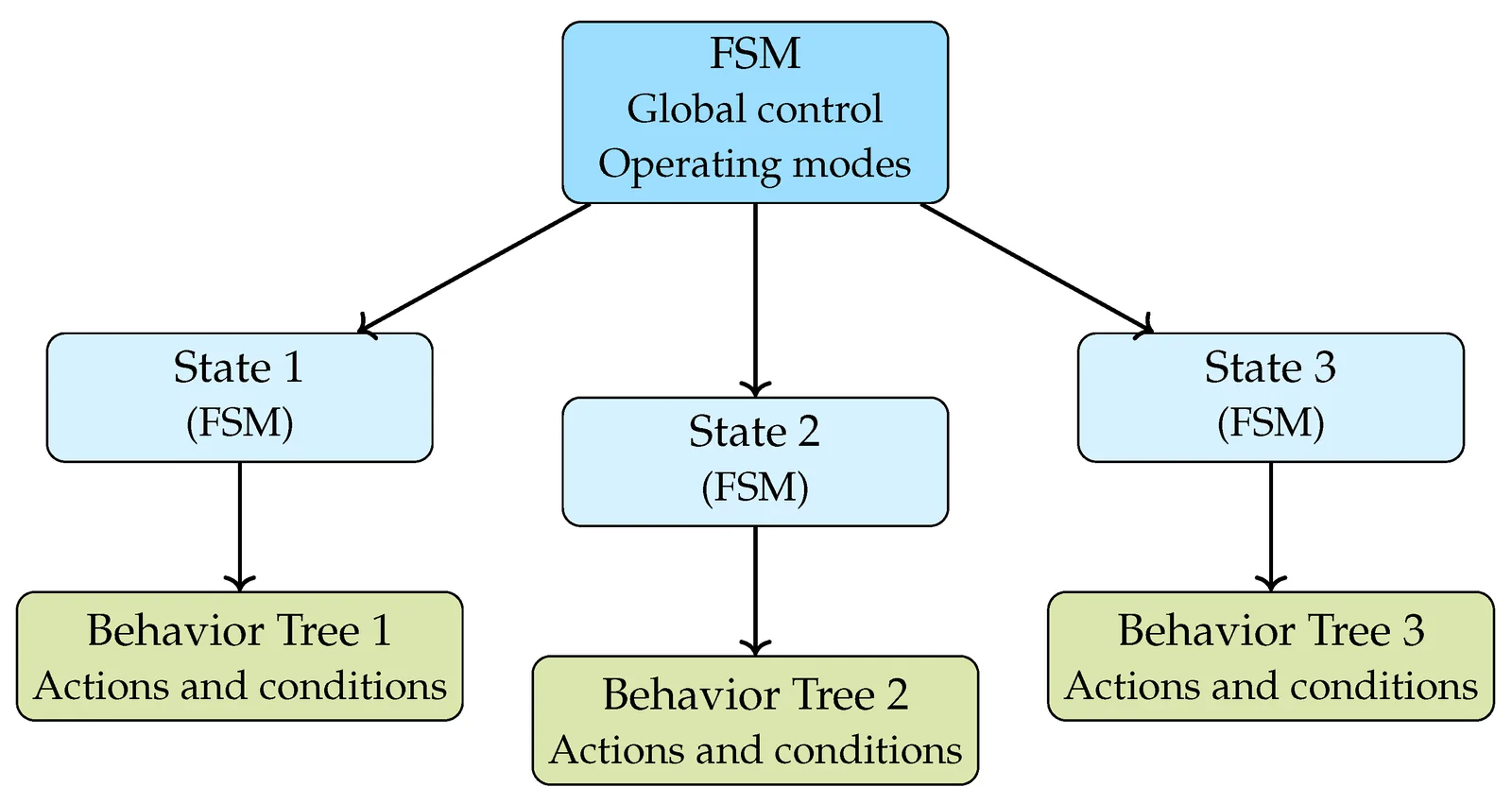
Hybrid hierarchical FSM–BT architecture.

The general organization of the proposed control architecture is illustrated in Figure 2. In this scheme, the FSM supervises the global operating states, while each state is associated with an independent BT responsible for executing the corresponding local behavior.

Figure 2 highlights the main architectural contribution of this work: the separation between global state supervision and local behavior execution. The FSM does not directly execute low-level actions; instead, it selects the current operating context. Once a state is active, the associated BT manages the detailed execution logic, including condition evaluation, action activation, and response to local sensory events.

This organization allows the system to preserve deterministic high-level supervision while enabling flexible local adaptation. The FSM does not directly implement motion trajectories, actuation profiles, or continuous control strategies. Instead, it defines the operational context in which such processes are permitted to occur. This separation reduces the complexity of the global controller and facilitates the future integration of additional rehabilitation modes or safety routines.

### 3.4. Global Supervision Based on FSM

The global supervision of the system is implemented through an FSM that operates as a high-level discrete coordinator. Its primary function is to manage the activation and deactivation of operating modes, arbitrate the execution flow between local behaviors, and ensure that safety constraints are respected throughout the rehabilitation process.

From a functional perspective, the FSM receives discrete events derived from three main sources: The first source corresponds to processed biomechanical indicators, such as movement thresholds, postural stability, gait phase, or user-interaction conditions. The second source corresponds to temporal events, including cycle completion, maximum execution times, waiting periods, or safety delays. The third source corresponds to external supervisory signals, such as therapist commands, user interface inputs, or system failure flags. These inputs are evaluated through logical conditions that determine the transition from one state to another.

**Figure 3:**
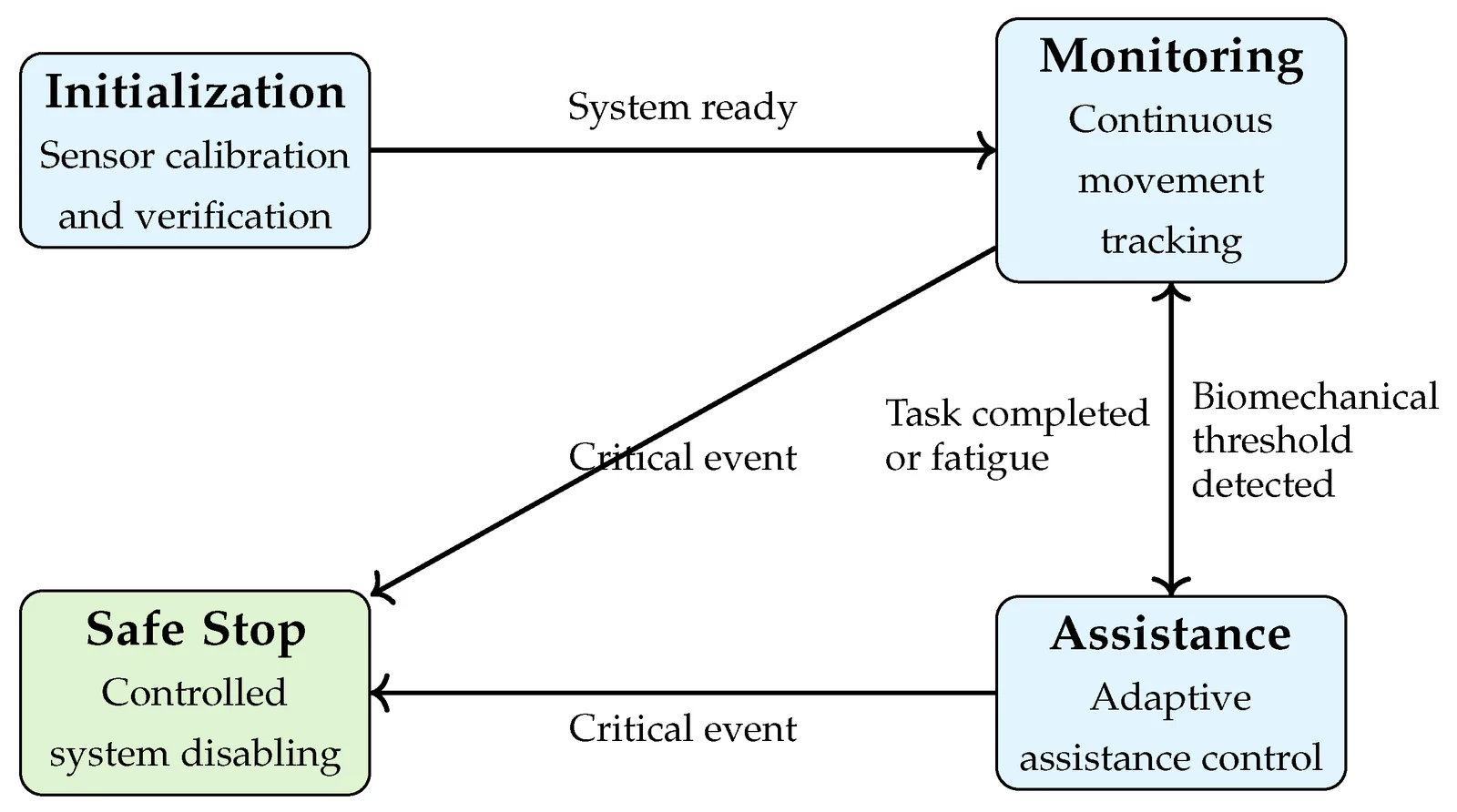
Global supervisory FSM with operational states, transition events, and self-transitions associated with monitoring and adaptive control.

A simplified representation of the global supervisory FSM is presented in Figure 3. This diagram shows the main operating states considered at the proposal level, together with representative transition events related to system readiness, biomechanical conditions, task completion, fatigue, and critical events. As observed in Figure 3, the FSM defines a deterministic supervisory structure in which normal operating states, such as monitoring and assistance, are clearly separated from the safe stop state. This organization allows safety-related events to interrupt the regular execution flow and redirect the system toward a controlled disabling condition. Therefore, the FSM provides a high-level mechanism for preserving operational coherence and safety during rehabilitation tasks.

### 3.5. Local Execution Based on Behavior Trees

The local execution of tasks is implemented using BTs, which act as reactive controllers responsible for the continuous evaluation of movement conditions, system state, and action execution. Each BT is strictly linked to a single FSM state and can only be executed while that state remains active. This restriction preserves coherence between the global supervisory decision and the local behavior being executed.

From a structural perspective, each BT is composed of control nodes, condition nodes, and action nodes. The root node acts as the entry point for the evaluation process, while control nodes, such as *Selector* and *Sequence* nodes, define the internal decision logic. Condition nodes evaluate biomechanical variables, system availability, safety constraints, and task-specific requirements. Action nodes execute atomic operations, such as generating assistance references, activating monitoring routines, updating control parameters, or requesting safe-stop actions.

**Figure 4:**
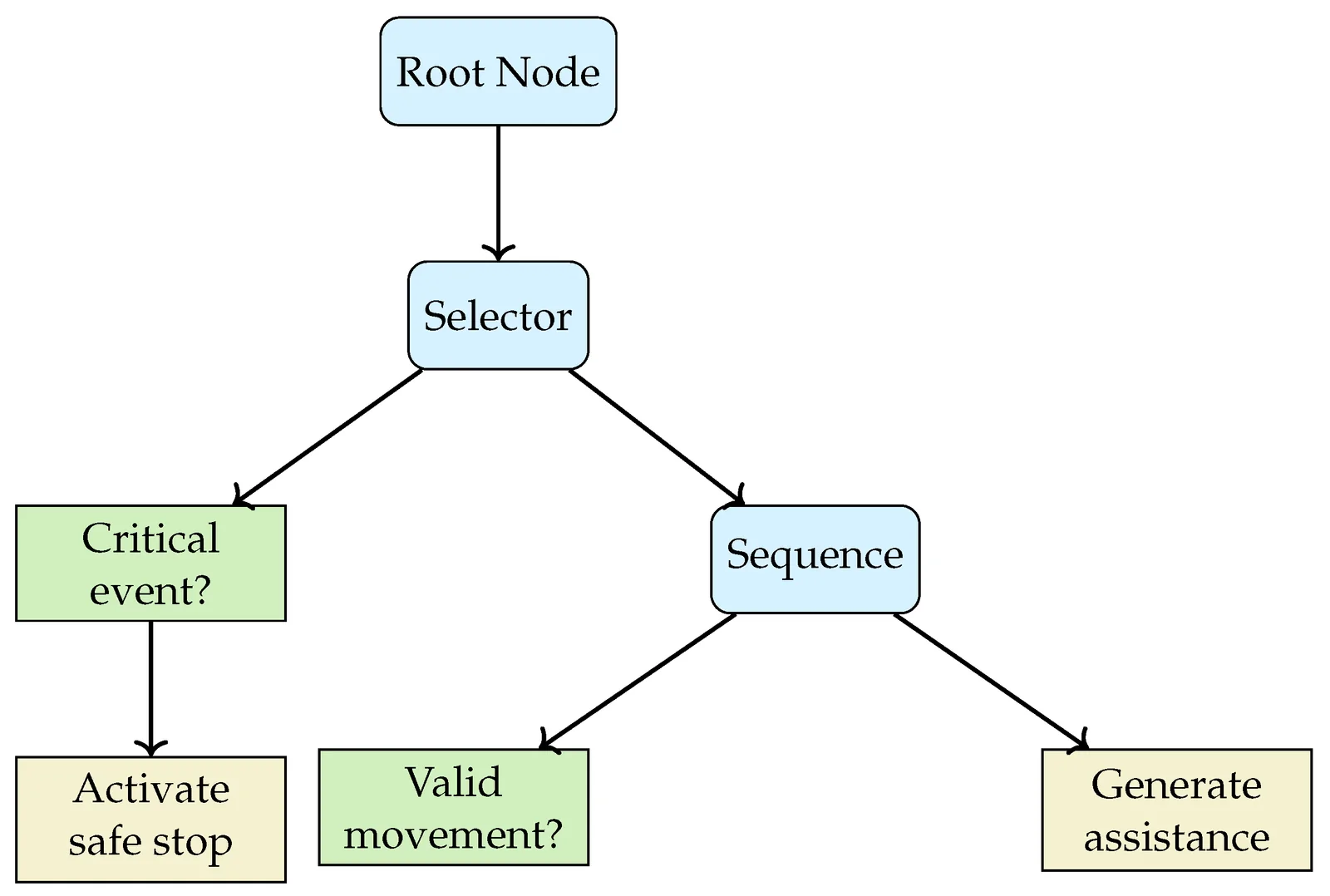
Internal structure of a Behavior Tree. The root node cyclically evaluates a selector that prioritizes critical events before executing the assistance sequence.

A representative internal structure of a BT is shown in Figure 4. This example illustrates how critical-event evaluation can be prioritized before the execution of an assistance sequence. Figure 4 shows that the BT structure enables local decision-making through the combination of control, condition, and action nodes. The selector node first evaluates whether a critical event is present. If such a condition is detected, the tree activates the safe-stop behavior. Otherwise, the sequence node allows the evaluation of movement validity and the subsequent generation of assistance. This structure supports reactive execution while preserving safety priorities at the local level.

From an operational perspective, each BT is evaluated cyclically at a fixed period $T_{BT}$, defined according to the monitoring and control requirements of the system. At each evaluation instant $t_{k}=kT_{BT}$, the tree is traversed from the root node to the leaf nodes. During this traversal, condition nodes are evaluated, action nodes are executed if enabled, and execution statuses are propagated back through the tree. The BT evaluation frequency is higher than the FSM update frequency, which can be expressed as $T_{FSM}\gg T_{BT}$.

### 3.6. FSM–BT Interaction and Execution Flow

When the FSM activates a specific state, the associated BT is initialized and enabled for cyclic evaluation. During this phase, the BT operates autonomously, evaluating local conditions and executing actions without direct intervention from the global supervisor.

The FSM does not alter the internal flow of the BT; instead, it monitors high-level events such as task completion, persistent error flags, safety violations, timeout conditions, or therapist commands.

If no transition condition is satisfied, the active BT continues its normal execution. If a transition condition is detected, the FSM requests a controlled termination of the current BT, ensuring that ongoing actions reach a safe configuration before the system enables the next state.

**Figure 5:**
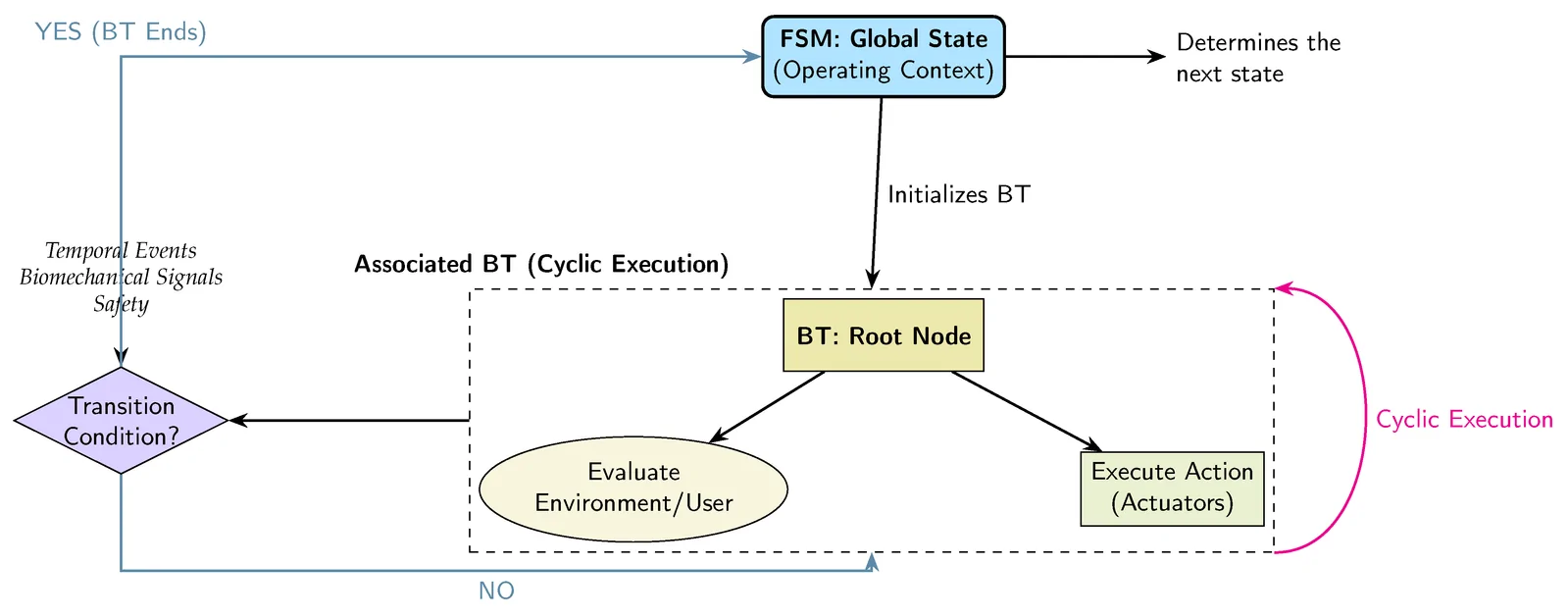
System architecture.

Figure 5 illustrates that the FSM determines the active operating context and initializes the corresponding BT. Once activated, the BT performs cyclic execution by evaluating the environment, user-related variables, and actuator actions. In parallel, transition conditions derived from temporal events, biomechanical signals, or safety constraints are monitored. If a transition condition is satisfied, the BT ends in a controlled manner and the FSM determines the next state. If no transition condition is detected, the BT continues its cyclic execution.

The complete execution flow is therefore characterized by a supervisor–executor cycle. The FSM defines the current operational context, enables the corresponding BT, monitors high-level transition events, and enforces safe transitions when required. The BT materializes the active context through reactive and adaptive behaviors, using sensory information to update local actions in real time. This interaction scheme enables the system to preserve coherence and safety while maintaining sufficient flexibility to respond to user-dependent variability.

### 3.7. Application of the Architecture to the SARA Rehabilitation Exoskeleton

In this manuscript, **SARA** denotes the project-specific lower-limb rehabilitation platform used as the target system for the proposed architecture. It is an eight-actuator passive–active exoskeleton concept intended to support supervised lower-limb rehabilitation. The control architecture is designed around four hip actuators (two per side), two knee actuators, and two ankle actuators. The SARA platform is under development; therefore, the present paper evaluates a software representation of its control interfaces rather than a physical prototype.

**Figure 6:**
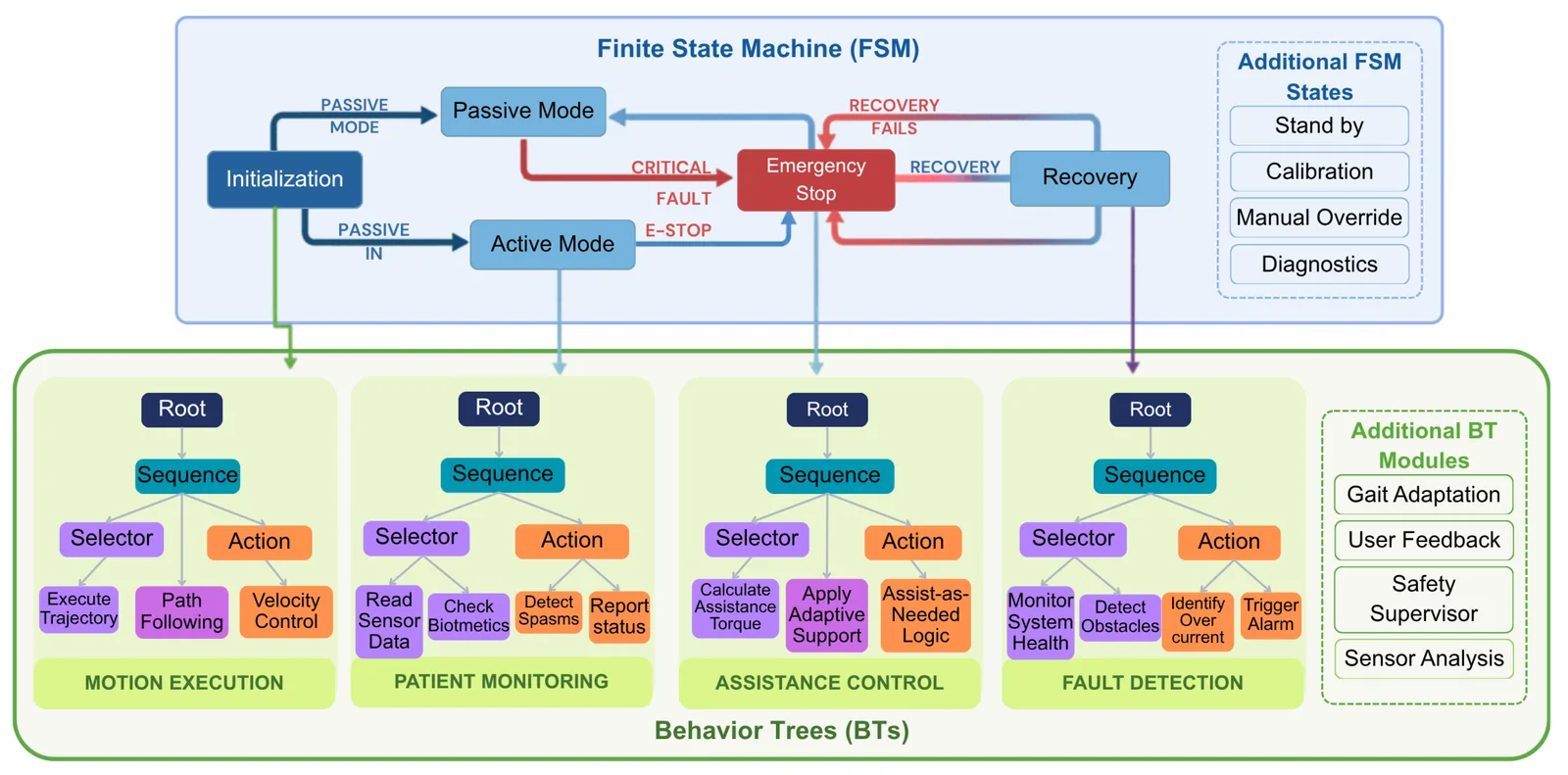
High-level hierarchical FSM–BT architecture mapped to rehabilitation functions. Additional FSM states and BT modules illustrate extensibility; the evaluated SIL subset is defined in Section 5.

The application of the hierarchical concept is illustrated in Figure 6. Global modes and safety escalation remain within the FSM layer, whereas motion execution, patient-state checks, assistance logic, and fault-recovery subroutines are implemented as BT modules. This allocation is intentionally asymmetric: adding a new local therapeutic routine should normally require adding or replacing a subtree, while changes that modify mutually exclusive global operating modes remain explicit FSM modifications.

**Figure 7:**
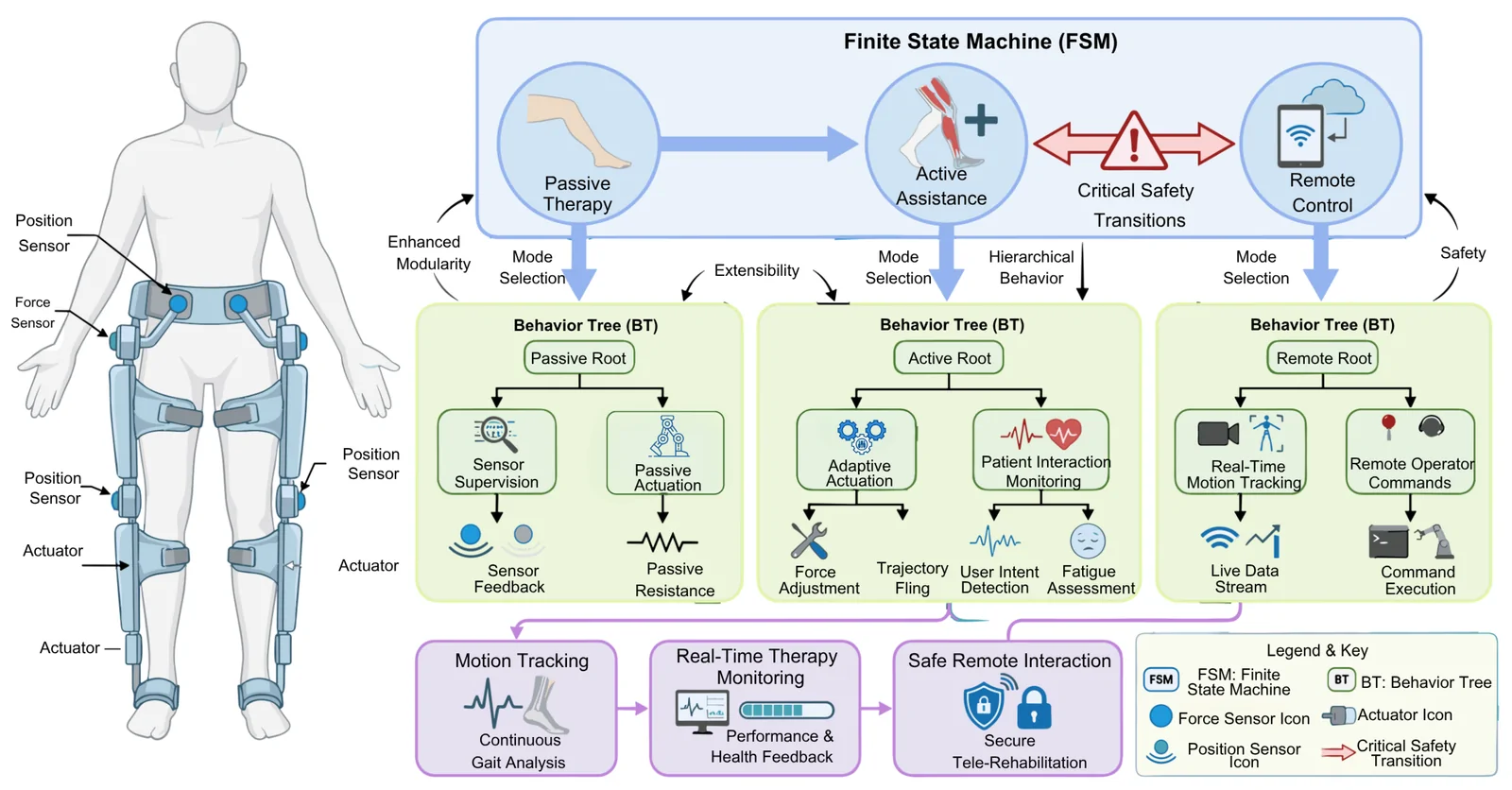
Conceptual application of the FSM–BT architecture to SARA. The remote-operation branch is shown only as a future architectural extension and is not part of the quantitative SIL validation.

Figure 7 retains the broader conceptual SARA application view originally developed for the project. It includes a remote-operation branch to show a possible architectural extension; however, **remote control and telerehabilitation were not quantitatively evaluated in this work**. Accordingly, remote-operation performance is not claimed in the Abstract, Keywords, Results, or Conclusions and remains an avenue for future work.

The two figures separate the evaluated architectural core from optional future functions. The remainder of this paper focuses on initialization, passive supervision, active assistance, passive–active mode switching, bounded recovery, and safety interruption.

## 4. Implementation

This section maps the formal architecture to the SARA target configuration and then defines the Software-in-the-Loop plant and low-level controller used for validation. The hardware architecture is included to specify intended interfaces only; all quantitative results reported in Section 5 were produced by the Python SIL model.

### 4.1. SARA Target Platform, Sensing Configuration, and SIL Plant Model

**Figure 8:**
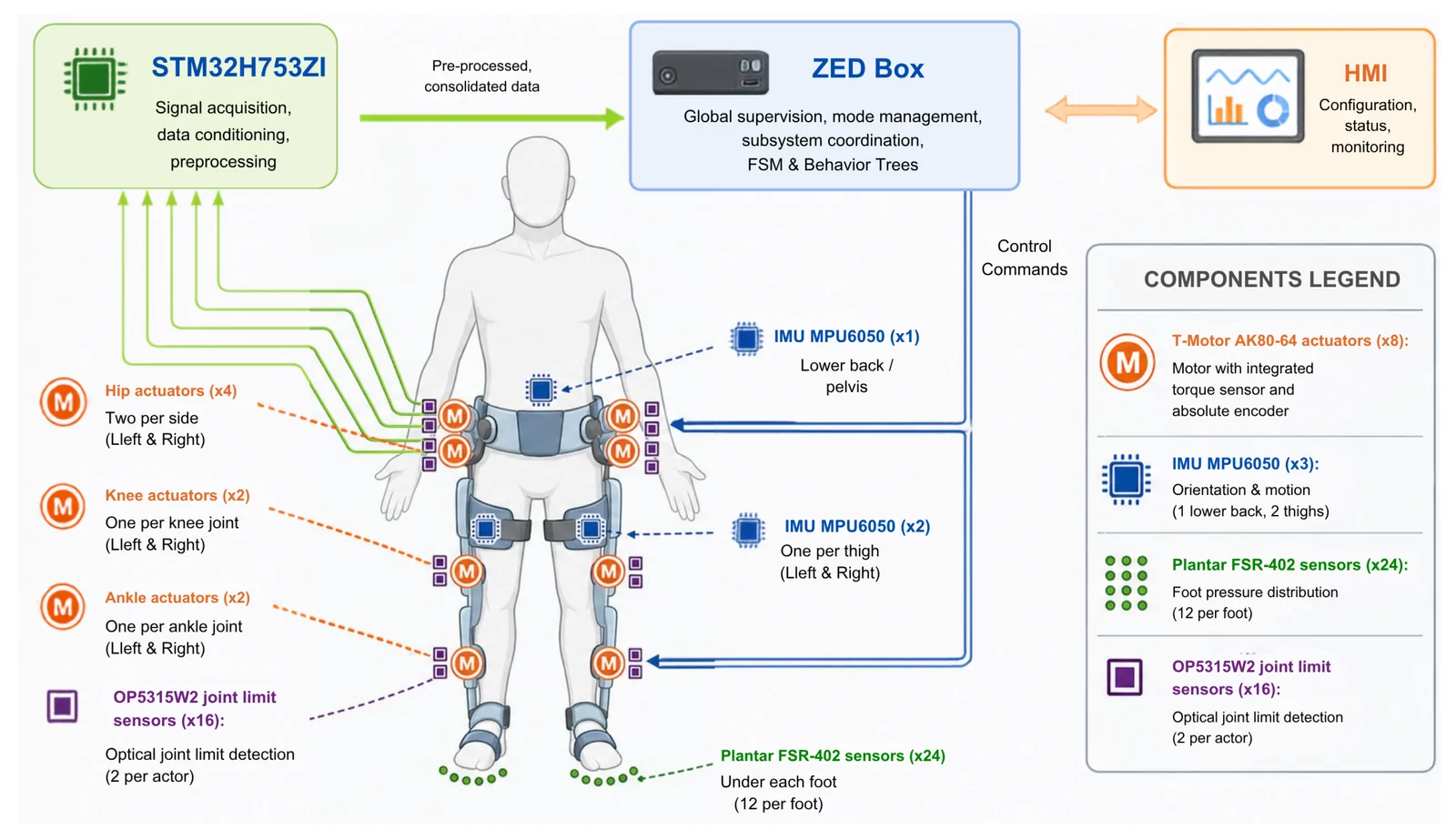
Intended SARA sensing, actuation, and processing architecture. The hardware components define the target deployment interfaces; no physical hardware measurements are reported in this study.

The intended SARA processing architecture is shown in Figure 8. The physical design assigns signal acquisition and preprocessing to an STM32H753ZI microcontroller (STMicroelectronics, Geneva, Switzerland) and supervisory processing to a ZED Box. The STM32H753ZI target is a 32-bit Arm Cortex-M7 device (Arm Limited, Cambridge, UK) operating at up to 480 MHz with 2 MB Flash and up to 1 MB RAM [36]. The ZED Box represents the intended Jetson-based high-level embedded-computing class [37]; an exact ZED Box compute-module configuration was not executed or benchmarked in this study. Consequently, no processor-load or hardware timing result depends on either target device.

The present SIL implementation does not instantiate a physical CAN, Ethernet, UART, or other bus between these processing levels. Instead, the STM32–ZED interface is represented as a bidirectional logical packet channel: the low-level side provides a consolidated sensor/status vector to the supervisor, and the supervisor returns mode, reference, assistance, and safe-stop commands. Each logical packet is subjected to the stochastic latency and packet-loss processes defined in Section 5.1. A physical protocol and its scheduling overhead will be selected and measured only during Hardware-in-the-Loop validation; therefore, the communication statistics reported here are explicitly software-model quantities rather than protocol benchmarks.

The SARA sensing architecture contains 59 sensing elements in the SIL configuration: eight actuator-integrated torque sensors, eight absolute joint encoders, three MPU6050 inertial measurement units (IMUs), twenty-four FSR-402 plantar-pressure sensors, and sixteen OP5315W2 optical joint-limit sensors. Table 6 summarizes how each group is represented in the simulation.

The exoskeleton plant is represented by eight decoupled second-order joint coordinates:

(6) $$I_{i}{\ddot{q}}_{i}+b_{i}{\dot{q}}_{i}=\tau_{i}+\tau_{ext,i},\;i=1,\ldots ,8,$$

where $I_{i}$ is the equivalent joint inertia, $b_{i}$ the viscous damping coefficient, $\tau_{i}$ the commanded actuator torque, and $\tau_{ext,i}$ a bounded generalized interaction torque. A complete musculoskeletal human model is not included; user/environment interaction is represented by $\tau_{ext,i}$ and by the simulated stability/load channels. This simplification is explicitly considered a limitation in Section 5.12. Table 7 reports the numerical plant and controller parameters used to reproduce the revised simulations.

The virtual torque saturation limits are 35, 35, 25, 25, 40, 40, 20, and 20 Nm for coordinates 1–8, respectively. Bilateral phase offsets are specified in the supplementary script. All trials start from $q_{i}\left(0\right)=0$ and ${\dot{q}}_{i}\left(0\right)=0$; the controller then tracks the same deterministic reference family while stochastic sensing, interaction, and communication realizations vary across Monte Carlo trials.

Joint tracking uses a torque-based proportional–derivative controller:

(7) $$\tau_{i}={\mathrm{sat}}_{\tau_{max,i}}\;\left[\alpha_{i}K_{p,i}\left(q_{d,i}-q_{m,i}\right)+\alpha_{i}K_{d,i}\left({\dot{q}}_{d,i}-{\dot{q}}_{i}\right)\right],$$

where $q_{m,i}=q_{i}+n_{q,i}$ is the noisy encoder measurement, $\alpha_{i}\in \left[0,1\right]$ is the assistance factor, and sat(·) enforces the virtual torque limit. In the comparative S2/S3 tests $\alpha_{i}=1$ for all eight coordinates so that supervisory architectures are compared under the same low-level controller. Patient-specific adaptation of $\alpha_{i}$ is therefore an architectural capability rather than a clinically validated result of this paper.

The reference used for the active-assistance benchmark is a smooth cyclic lower-limb trajectory:

(8) $$q_{d,i}\left(t\right)=A_{i}\sin\left(2\pi f_{i}t+\phi_{i}\right),$$

with joint-dependent amplitudes, frequencies, and bilateral phases specified in the accompanying Python script. Recent rehabilitation-control studies emphasize that low-level disturbance rejection and joint tracking are themselves substantial control problems [33,34,35]; in contrast, the present contribution focuses on the supervisory architecture and, therefore, intentionally uses the same transparent PD law for every architectural baseline.

### 4.2. Implementation of the Global FSM

**Figure 9:**
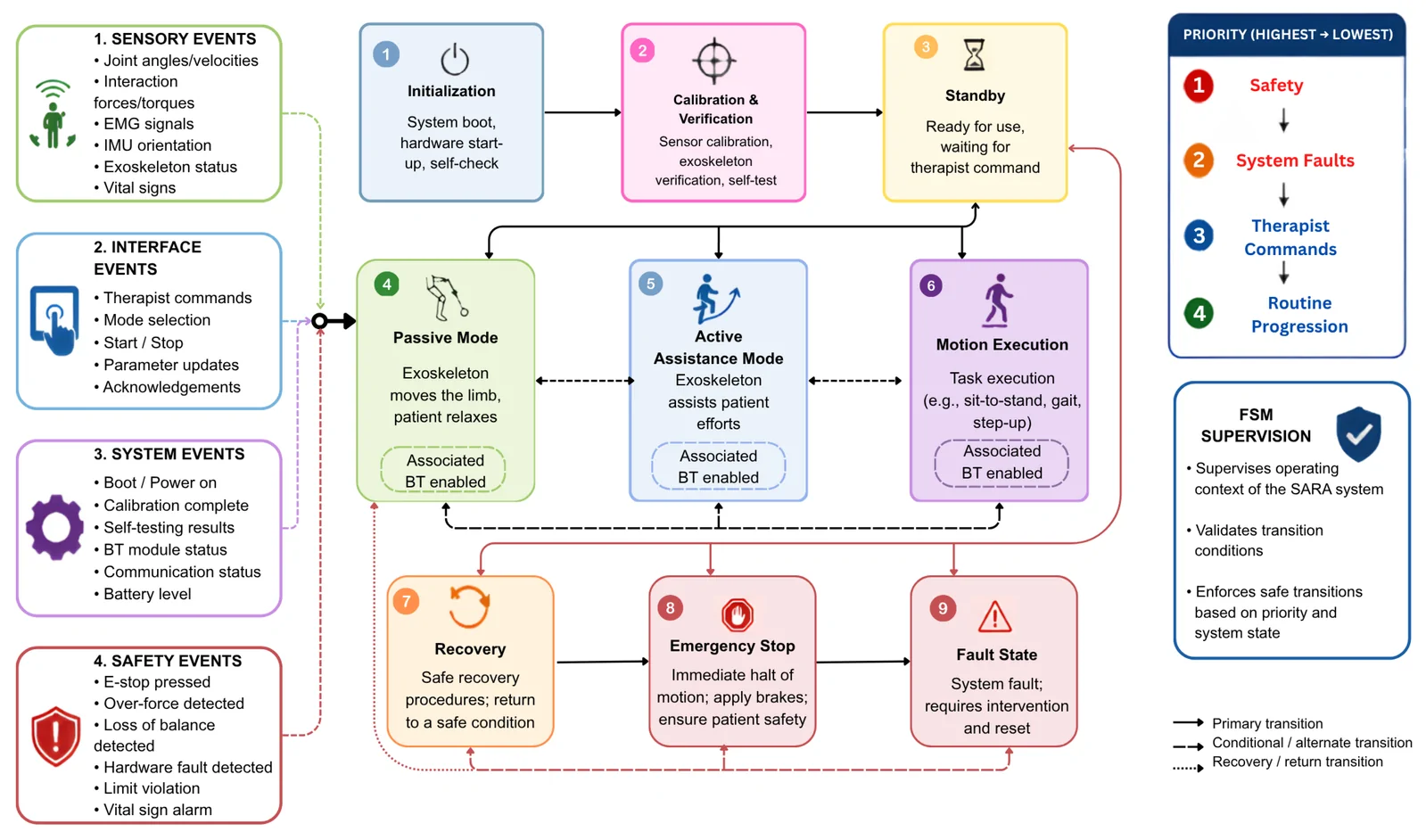
Implemented global FSM for the SARA rehabilitation system.

The SARA implementation instantiates the supervisory formulation of Section 3 as the nine-state FSM shown in Figure 9. The FSM authorizes operating modes, applies event priority, and enables the corresponding BT; it does not generate continuous joint commands, which are produced by the low-level controller in Equation (7). The figure summarizes the application-specific states, event sources, safety escalation, and BT associations without repeating the generic FSM–BT execution logic introduced earlier.

The nine states are *Initialization*, *Calibration and Verification*, *Standby*, *Passive Mode*, *Active Assistance Mode*, *Motion Execution*, *Recovery*, *Emergency Stop*, and *Fault State*. Initialization and calibration validate the modeled resource/status conditions before entry to *Standby*. The passive and active states represent the mutually exclusive therapeutic contexts; *Motion Execution* activates the corresponding task BT. Recoverable persistent events are routed to *Recovery*, critical events to *Emergency Stop*, and unrecoverable conditions to *Fault State*.

Four event classes drive the implementation: sensory, interface, system, and safety events. Continuous variables are converted to logical guards before reaching the FSM. Events are ordered by priority as safety events, persistent system faults, therapist/interface commands, and routine-progression events. Thus, a simultaneous joint-limit violation and therapist command is resolved in favor of the safety transition. This event-priority mechanism is the rehabilitation-specific arbitration rule used in the S3 and S4 simulations.

The following table summarizes the main event categories used by the implemented FSM.

Table 8 makes the abstraction boundary explicit: the FSM consumes logical events rather than raw sensor samples. The operational states *Passive Mode*, *Active Assistance Mode*, and *Motion Execution* activate their associated BT modules; BT completion, persistent failure, or escalation is returned to the FSM as an event. This preserves a single global operating context, while local adaptation remains inside the BT layer.

### 4.3. Implementation and Design Criteria of the Behavior Trees

The BT layer materializes the behavior authorized by the current FSM state. Each node was designed according to four explicit criteria. First, *atomicity*: an Action node performs one functional operation (for example, generating a reference or enabling assistance). Second, *separation of verification and execution*: side-effect-free checks are Condition nodes, whereas operations that modify system variables are Action nodes. Third, *safety-first ordering*: safety and validity conditions are evaluated before the action that they guard. Fourth, *local recoverability*: a recoverable transient condition is handled by a bounded recovery branch before the event is escalated to the global FSM.

The procedure for extending a BT is likewise explicit. A new function is first classified as either a *global-mode change* or an *in-mode behavior*. Global-mode changes modify the FSM; in-mode behaviors are inserted into the BT associated with the current mode. The developer then (i) defines the node input variables, output variables, and return status; (ii) classifies the node as Condition, Action, Control, or Recovery; (iii) defines the safety guard and timeout; (iv) defines whether a failure is locally recoverable; (v) inserts the node/subtree at the smallest affected branch; and (vi) reruns the structural and SIL regression tests. This procedure is the basis of the scalability study reported later.

Each functional leaf can be represented as

(9) $$N_{i}=\left(I_{i},O_{i},\sigma_{i},\theta_{i}\right),$$

where $I_{i}$ and $O_{i}$ denote inputs and outputs, $\sigma_{i}\in \left\{\mathrm{Success},\mathrm{Failure},\mathrm{Running}\right\}$ is the return status, and $\theta_{i}$ contains node-specific thresholds, timeout, and recovery parameters.

The implemented BT layer contains 51 nodes distributed across three representative trees. The System Initialization BT contains 15 nodes; the Therapy Configuration BT and Exercise Execution BT contain 18 nodes each. Table 9 provides the node-size breakdown.

**Figure 10:**
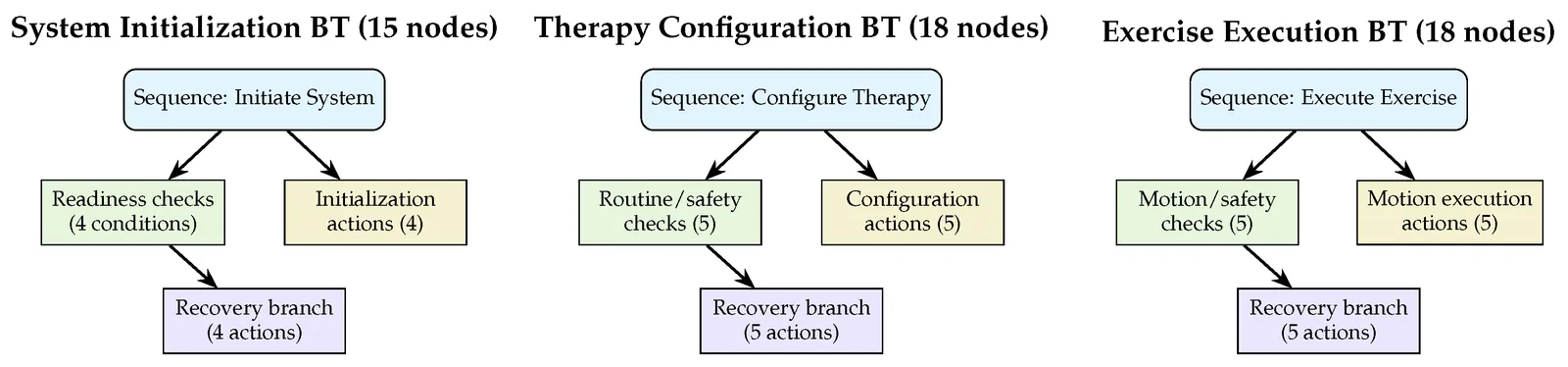
Simplified vector representation of the implemented BT modules and corrected root-control labels. Detailed node counts are provided in Table 9.

Figure 10 provides a simplified vector schematic of the three implemented BT modules so that their root-control labels and recovery structure remain readable at normal page scale.

The System Initialization BT checks communication availability, sensor consistency, actuator readiness, and posture validity before executing initialization, calibration, actuator enabling, and monitoring actions. The Therapy Configuration BT verifies routine selection, parameter validity, joint-limit compatibility, plantar-load distribution, and user stability before loading the routine and calculating safety limits. The Exercise Execution BT validates the initial posture and safety conditions before generating the reference, enabling assistance, monitoring execution, updating the reference, and checking completion.

#### Failure Recovery and Escalation

Failures are divided into locally recoverable and globally critical events. In the SIL benchmark, a local branch is permitted up to $R_{BT}=2$ corrective attempts. Examples include retrying a transient communication operation, revalidating a noisy sensor condition, or rechecking posture. If those attempts fail, the hybrid architecture escalates the event once to the FSM *Recovery* state. A critical condition bypasses routine recovery and requests *Emergency Stop* directly. Table 10 presents the recovery and escalation logic of the proposed architecture, showing the local BT responses to different failure conditions and the corresponding FSM-level responses for persistent or critical events.

The escalation chain can be summarized as

(10) $$\mathrm{Local}\;\mathrm{Failure}\to \mathrm{BT}\;\mathrm{Recovery}\to \left\{\begin{array}{cc} \mathrm{Resume}, & \mathrm{if}\;\mathrm{recovered},\\ \mathrm{FSM}\;\mathrm{Recovery}/\mathrm{Fault}, & \mathrm{if}\;\mathrm{persistent},\\ \mathrm{Emergency}\;\mathrm{Stop}, & \mathrm{if}\;\mathrm{critical}. \end{array}\right.$$

This separation prevents every transient anomaly from becoming a global state transition while retaining explicit FSM authority when local recovery is insufficient.

### 4.4. Integration of Sensing–Control–Actuation and Safety Mechanisms

The controller uses the sensing channels at two abstraction levels. Continuous variables such as joint angle, torque, orientation, and plantar-load distribution are first converted into local BT conditions. The FSM receives only supervisory events, including *mode request*, *task completed*, *recovery failed*, *communication timeout*, and *critical safety event*. This separation avoids direct coupling between raw sensor samples and global state transitions.

The SIL safety thresholds are intentionally explicit: joint-limit proximity is declared when the estimated angle enters a 5° safety margin; unexpected resistance is declared above 12 Nm; sensor inconsistency above 5° between the compared virtual orientation channels; user instability above a 10° orientation deviation; and communication timeout above 50 ms or after three consecutive lost packets. A safety condition must remain true for four consecutive BT evaluations before ordinary escalation, except an externally asserted emergency-stop event, which is processed immediately. These values are simulation thresholds used for controller-level testing and are not proposed as clinically validated limits. The selected thresholds were defined as engineering parameters for the SIL benchmark to distinguish nominal variability from abnormal conditions. They were selected based on the simulated noise, controller behavior, and fault conditions to ensure reproducible detection. These values are simulation parameters and should not be interpreted as clinically validated or hardware-certified safety limits.

The safety response reported in this paper ends when the software supervisor reaches the *Emergency Stop* state. It therefore contains unsafe-condition sampling/detection and the corresponding software decision. A separate simulated communication delay is reported for dispatch of the stop command. Actuator inhibition time, brake engagement, human–exoskeleton dynamics, and the mechanical time needed to reach a safe physical condition are **not modeled**. This distinction prevents the SIL response time from being interpreted as a physical stopping-time measurement.

## 5. Results

### 5.1. Software-in-the-Loop Setup and Reproducibility

The proposed architecture was validated exclusively in a custom Software-in-the-Loop environment developed in Python 3.13.5. NumPy 2.3.5 was used for numerical computation, Pandas 2.2.3 for tabulation and post-processing, Matplotlib 3.10.8 for figures, and SciPy 1.17.0 for statistical testing. No Hardware-in-the-Loop execution, STM32/ZED execution, physical actuator test, or human-subject experiment is part of these results.

The eight joint coordinates described by Equation (6) were integrated using a fixed step Δt=0.01 s (100 Hz). The FSM update period was $T_{FSM}=100$ ms (10 Hz), whereas BTs were ticked every $T_{BT}=20$ ms (50 Hz). Every trial lasted 8 s. Thirty Monte Carlo trials were run for each of four scenarios and each of three architectures, producing 120 trials per architecture and 360 comparative trials. A fixed pseudo-random seed of 2026 generated common random realizations for all architectures so that pairwise statistical comparisons used matched disturbances.

Table 11 summarizes the main simulation conditions. The normal latency distribution was truncated at zero. The full 59-channel sensing configuration was represented at the interface level; encoder noise directly affected tracking feedback, whereas the remaining virtual sensor groups generated the supervisory conditions described in Section 4.

The Monte Carlo variability originates from encoder and interaction-torque noise, communication latency and packet loss, the phase of events relative to the FSM/BT clocks, the type and phase of the injected S4 abnormal condition, and the number of attempts required by transient recovery. These stochastic sources define the trial-to-trial variability used in the Monte Carlo analysis.

### 5.2. Validation Scenarios and Failure Injection

The four scenarios are retained from the original study and are summarized in Table 12. S1 evaluates passive supervision. S2 evaluates active joint tracking and local recovery. S3 stresses mode arbitration by introducing passive–active requests at random phases; 20% of S3 trials additionally contain a simultaneous routine-completion/mode-request event to test global-mode consistency. S4 injects one of five unsafe conditions (joint-limit proximity, unexpected resistance, sensor inconsistency, user instability, or communication timeout) for a 150 ms interval at a random phase relative to the local evaluation clock.

For S4, the injected values were set beyond the software thresholds: joint-limit proximity inside the 5° safety margin, resistance at 14 Nm (>12 Nm), sensor disagreement at 7° (>5°), orientation instability at 12° (>10°), or communication timeout at 60 ms/three consecutive packet losses. These values are test stimuli rather than clinical limits.

### 5.3. Outcome Definitions and Statistical Analysis

A trial is classified as successful only if the required task completes without an unresolved recovery, global-mode inconsistency, or missed S4 software stop criterion. A rejected transition whose guard is false is not counted as a controller failure; it is a correct safety rejection. Recovery success is

(11) $$S_{rec}=\frac{N_{recovered}}{N_{recovery\;required}}\times 100\%.$$

Global-mode consistency is the fraction of S3 trials in which the requested mutually exclusive mode is unambiguously represented after event arbitration. Overall trial success is

(12) $$S_{trial}=\frac{N_{successful\;trials}}{N_{trials}}\times 100\%.$$

For tracking, the joint error is

(13) $$e_{i}\left[k\right]=q_{d,i}\left[k\right]-q_{i}\left[k\right].$$

The global eight-DOF RMSE and MAE are

(14) $$\mathrm{RMSE}=\sqrt{\frac{1}{8N_{t}}\sum_{i=1}^{8}\sum_{k=1}^{N_{t}}e_{i}{\left[k\right]}^{2}},$$

(15) $$\mathrm{MAE}=\frac{1}{8N_{t}}\sum_{i=1}^{8}\sum_{k=1}^{N_{t}}\left|e_{i}\left[k\right]\right|.$$

The reported NRMSE is the mean of the per-joint RMSE normalized by that joint’s peak-to-peak reference range. Packet loss is calculated as $100N_{lost}/N_{total}$.

Ninety-five-percent Wilson intervals are reported for overall success proportions. Because each architecture uses the same random trial realizations, paired exact McNemar tests are used for binary outcomes, and a paired Wilcoxon signed-rank test is used for safety-response timing. Statistical significance is interpreted at α=0.05.

### 5.4. FSM Supervision and Passive–Active Mode Switching

For the proposed FSM–BT architecture, a valid S3 mode request required 70.1 ms on average from request generation to the first tick of the newly enabled BT. This value includes the phase-dependent wait to the next 10 Hz FSM evaluation, the phase to the first 50 Hz BT tick, and one simulated communication-latency sample. This metric is a software scheduling quantity and is not interpreted as a physical hardware transition time.

The hybrid controller maintained 100% global-mode consistency in the 30 S3 trials. The BT-only baseline achieved 90.0% because three deliberately simultaneous completion/request events resulted in an ambiguous global-mode selection in the monolithic tree. The FSM-only baseline also maintained explicit global-mode consistency, but its lower local recovery capability reduced overall S2/S3 task success, as discussed below.

### 5.5. Behavior-Tree Execution and Failure Recovery

Across the 32 matched trials in which a transient recovery was required, the hybrid recovered 93.75%, the BT-only controller 81.25%, and the FSM-only controller 59.38%. The hybrid and BT-only architectures both benefited from 50 Hz local reevaluation; however, only the hybrid could escalate an unresolved local transient to one explicit FSM recovery attempt without immediately terminating the task. Relative to FSM-only, the hybrid recovery improvement was significant by exact McNemar test ($p=9.77\times 10^{-4}$). The recovery difference relative to BT-only was directionally favorable but not statistically significant (p=0.125), which is reported to avoid overstating the evidence.

The failures therefore have an interpretable origin. FSM-only failures were dominated by transients that required more than the single global recovery opportunity allowed in the equivalent policy. BT-only failures were caused by transients that exceeded its bounded local recovery capacity and by the lack of an explicit global recovery/mode state in simultaneous-event S3 trials. Hybrid failures occurred only when the transient exceeded both local and one global recovery opportunity.

### 5.6. Software Safety Response

The S4 metric is strictly a controller-level timing quantity. Let $t_{fault}$ denote fault injection, $t_{det}$ the first instant at which the required four unsafe BT evaluations have been accumulated, $t_{stop}$ the instant at which the supervisory state becomes *Emergency Stop*, and $t_{dispatch}$ the simulated communication dispatch of the stop command. Unsafe-condition detection therefore ends at $t_{det}$. The controller decision is executed in the same scheduler cycle, so the modeled decision delay $t_{stop}-t_{det}$ is zero at the simulation time resolution. Command transmission is represented separately by the sampled latency *L*. Actuator-drive inhibition, braking, and mechanical settling are not modeled. Thus,

(16) $$T_{software}=\left(t_{stop}-t_{fault}\right)\times 1000.$$

The simulated command-dispatch time is

(17) $$T_{dispatch}=T_{software}+L.$$

Neither quantity includes actuator inhibition, braking, or mechanical settling.

**Figure 11:**
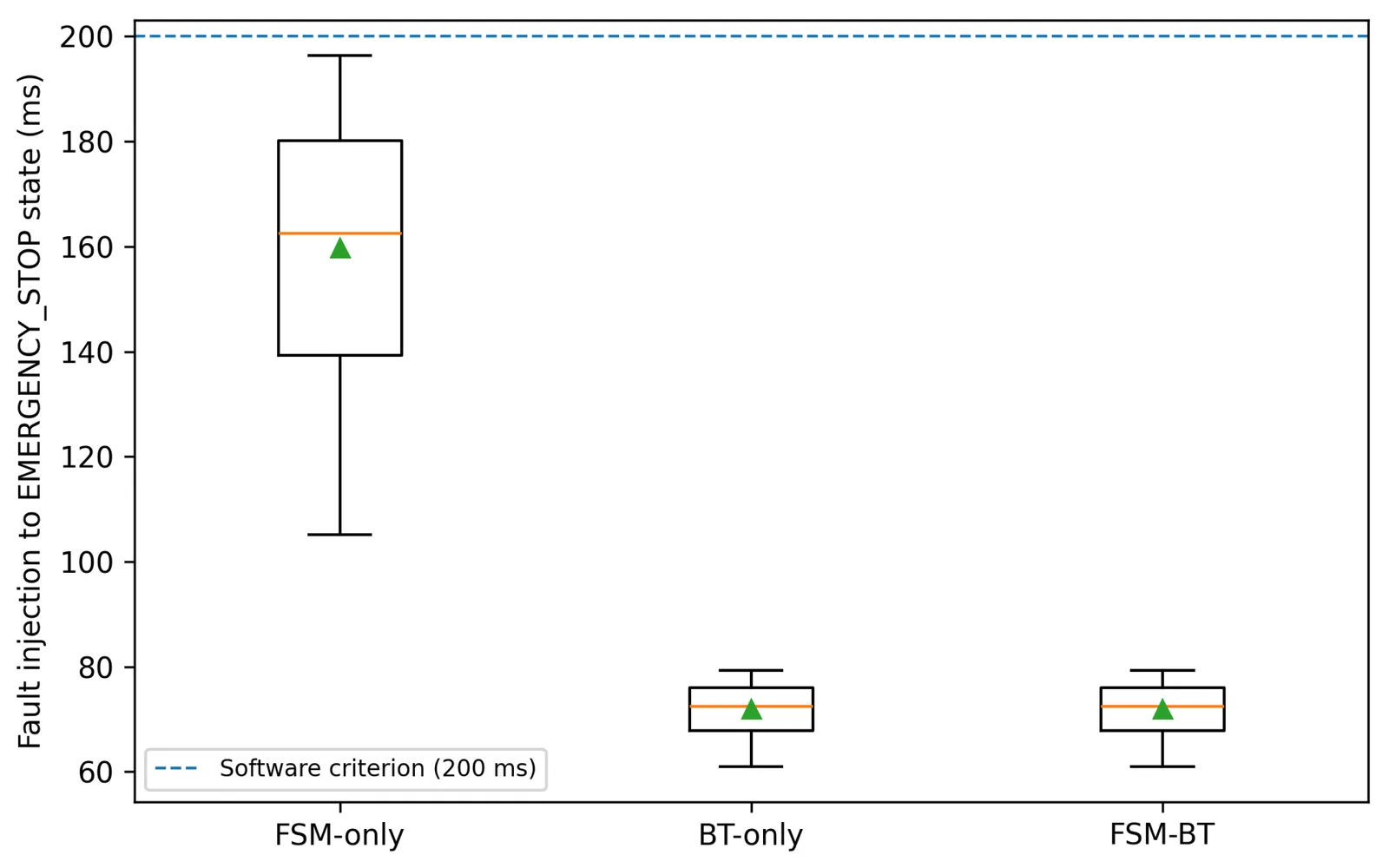
Distribution of software-level time from S4 fault injection to the *Emergency Stop* state. The 200 ms line is a SIL acceptance criterion, not a physical stopping-time requirement.

As shown in Figure 11, the software-level fault-injection time for reaching the Emergency Stop state remained below the 200 ms SIL acceptance criterion for all three control policies. The FSM-only approach exhibited substantially greater variability, whereas the BT-only and FSM–BT approaches showed lower and more consistent response times.

The orange line indicates the median, while the green triangle indicates the mean. The box represents the interquartile range, and the whiskers indicate the observed range. The dashed blue line represents the 200 ms SIL acceptance criterion, not a physical stopping-time requirement.

The FSM–BT mean $T_{software}$ was 71.9 ms (SD 5.27 ms; 95% CI 70.0–73.9 ms), and the mean command-dispatch value was 81.0 ms. BT-only produced the same software timing because it used the same four-confirmation 50 Hz local cadence. FSM-only required 159.7 ms on average (SD 26.34 ms; 95% CI 149.8–169.5 ms) because its safety condition was evaluated at the 10 Hz state-machine cadence. The paired hybrid-versus-FSM-only difference was significant ($p=9.31\times 10^{-10}$, one-sided Wilcoxon test).

All three policies satisfied the 200 ms software criterion in the generated S4 trials. Consequently, the safety comparison supports a timing advantage of fast local reevaluation over FSM-only supervision, but it does not demonstrate the mechanical safety of the physical exoskeleton.

### 5.7. Active-Assistance Tracking Performance

Because all three supervisory architectures use exactly the same low-level plant and PD controller, their tracking distributions are intentionally identical. This design isolates architectural effects from low-level motion-control effects. Across the 60 hybrid S2/S3 trials, the global eight-DOF RMSE was 2.424±0.008° (mean ± SD), MAE was 1.764±0.009°, and NRMSE was 7.49±0.04%. The mean peak commanded torque was 9.20 Nm. No hardware torque capability is inferred from this value because it belongs to the simplified SIL model (See Table 13).

**Figure 12:**
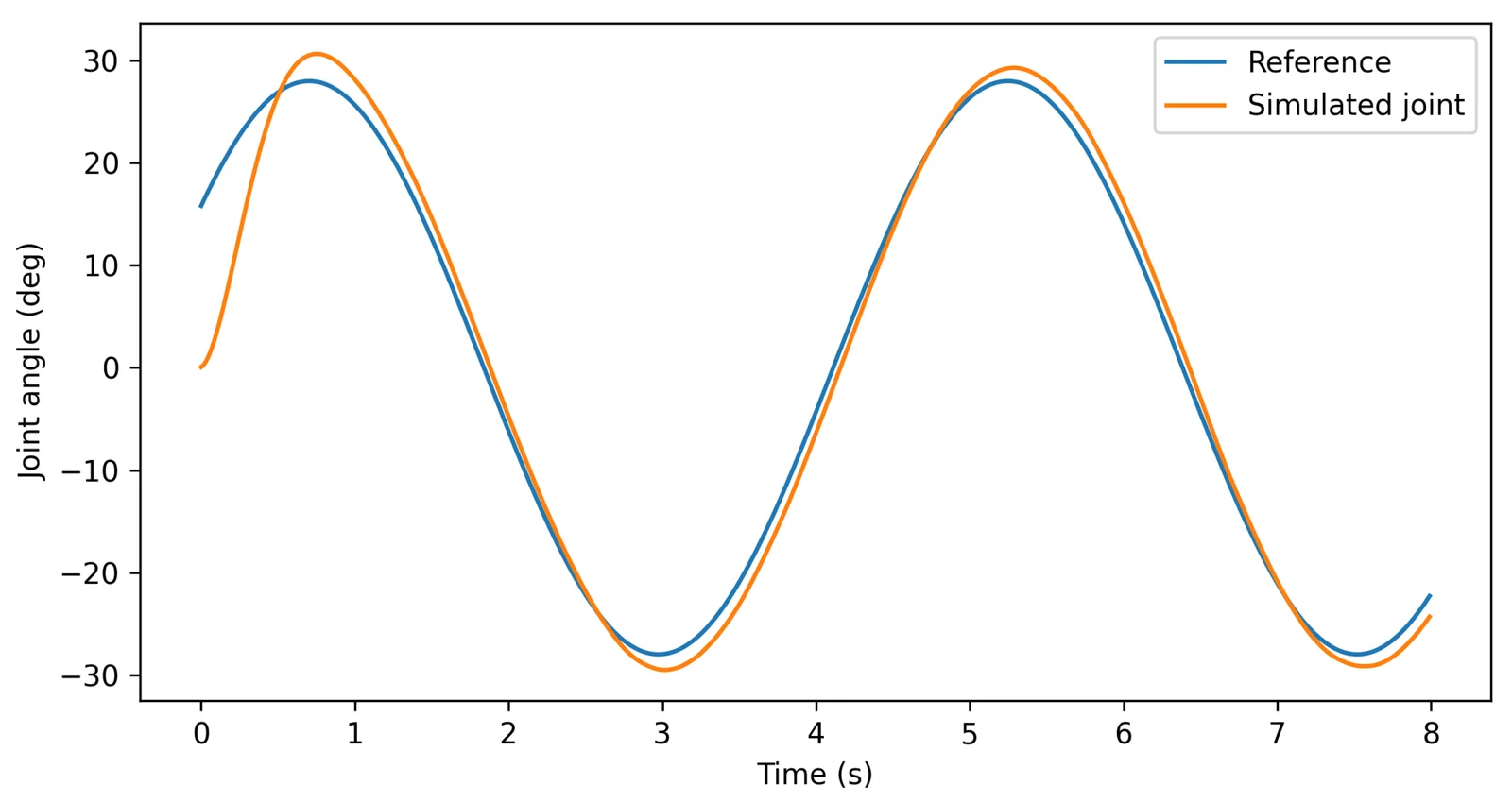
Representative reference and simulated joint trajectory for one active-assistance trial.

The representative signals and RMSE histogram provide both time-domain and distributional evidence for the reported tracking statistics (See Figure 12).

### 5.8. Passive-Mode Supervision and Communication Model

**Figure 13:**
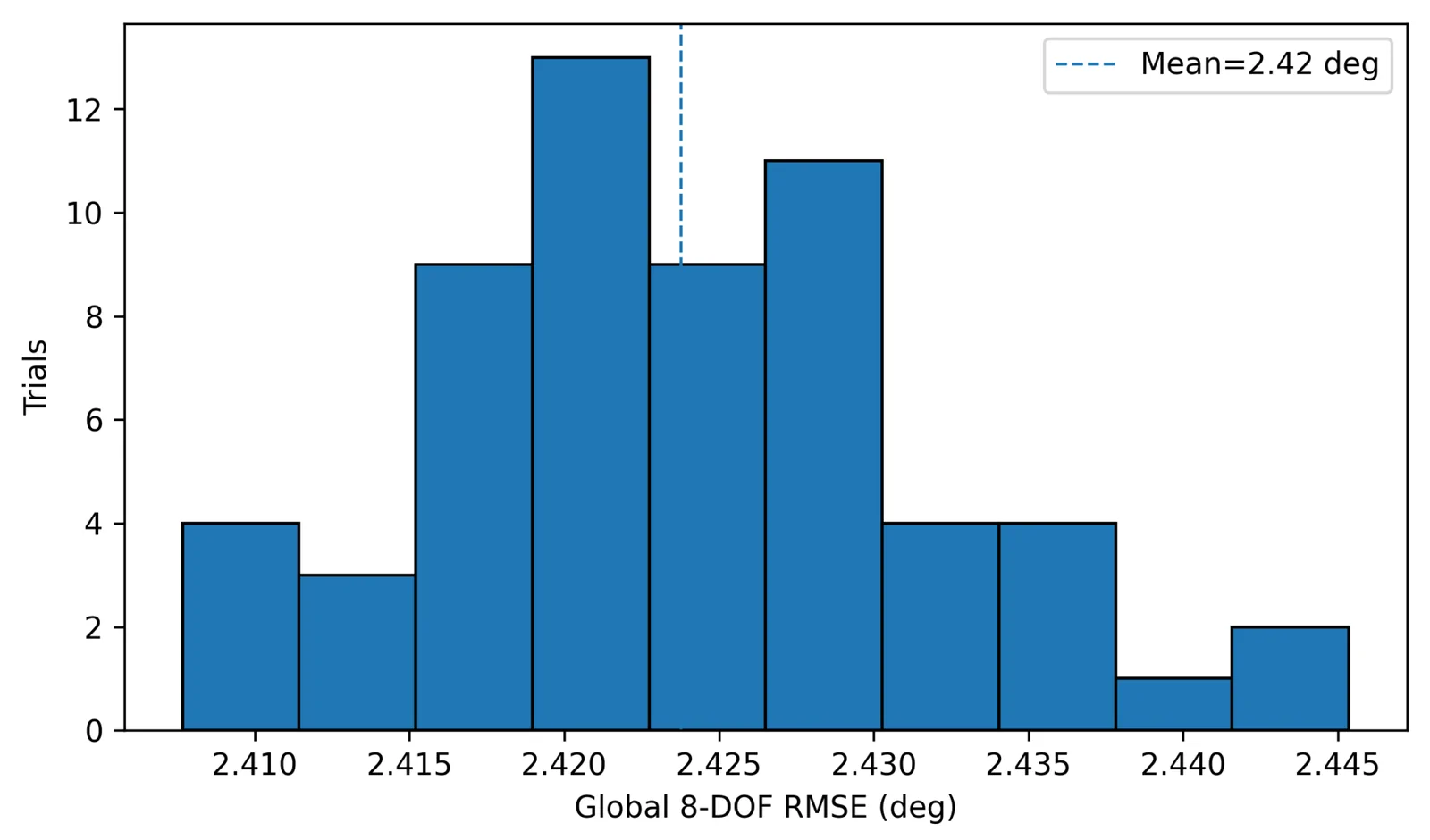
Distribution of the global eight-DOF RMSE across the 60 hybrid S2/S3 tracking trials.

In S1, the hybrid completed all 30 trials successfully. Five trials contained a transient recoverable anomaly, and all five were recovered without leaving the authorized passive mode. For the active tracking scenarios, the distribution of the global eight-DOF RMSE across the S2/S3 trials is shown in Figure 13. No physical passive-resistance measurement is claimed because passive user interaction is represented only by the generalized interaction torque and supervisory sensor conditions.

Communication is modeled, not measured. Across the 120 common hybrid trials, the mean latency was 9.002 ms, the largest sampled latency was 17.09 ms, and the simulated packet-loss fraction was 0.775%. These statistics follow from the specified truncated Gaussian latency and Bernoulli loss processes and should not be interpreted as CAN, Ethernet, STM32, or ZED Box measurements. No STM32 or ZED Box utilization percentage is reported because no hardware execution was performed.

### 5.9. Baseline Comparison and Statistical Evidence

**Figure 14:**
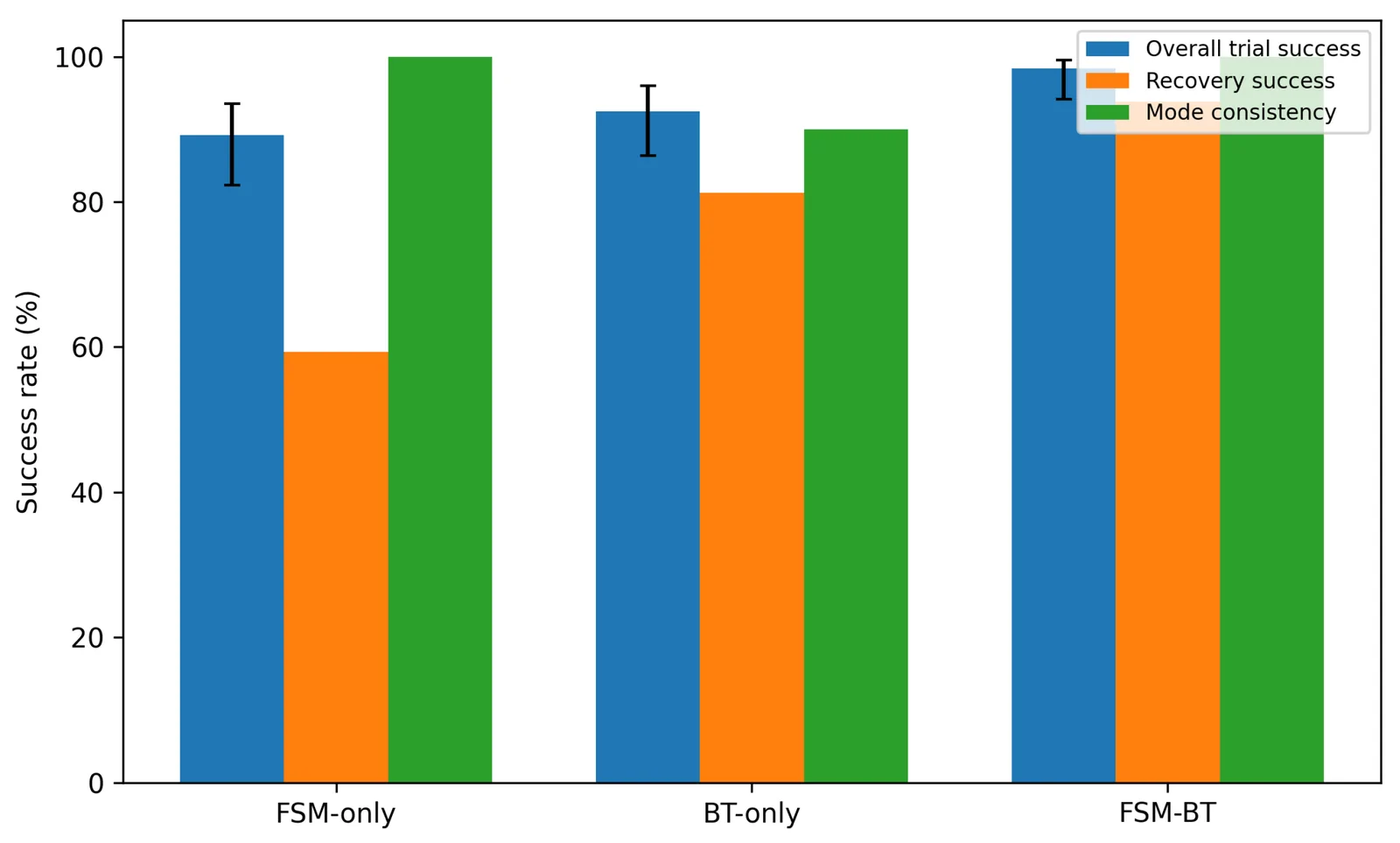
Matched SIL comparison of overall trial success, recovery success, and global-mode consistency. Error bars on overall success show 95% Wilson intervals.

Figure 14 and Table 14 provide the comparison among the three functionally equivalent supervisory policies. The hybrid achieved 98.3% overall success (95% Wilson CI 94.1–99.5%), compared with 89.2% (82.3–93.6%) for FSM-only and 92.5% (86.4–96.0%) for BT-only. Exact paired McNemar tests found the hybrid improvement to be significant relative to FSM-only ($p=9.77\times 10^{-4}$) and BT-only (p=0.0156).

The comparison also clarifies what the hybrid does *not* improve. BT-only and FSM–BT have identical configured local safety cadence, so no timing advantage is observed between them in S4. Likewise, tracking is identical because the same low-level controller is deliberately shared. The hybrid benefit appears in the combination of explicit global-mode semantics and local recovery rather than in an artificially different plant or motion controller.

### 5.10. Scalability and Structural Extension Study

To test the original scalability claim directly, four functionally equivalent structural encodings were constructed: FSM-only, hierarchical state machine (HSM-only), BT-only, and the proposed FSM–BT. The baseline hybrid contains nine FSM states, seventeen FSM transitions, and 51 BT nodes. A new therapeutic routine is represented as an eight-leaf local behavior plus the structural attachment required by the corresponding representation. An *edit operation* is one state, transition, node, or existing attachment that must be added or modified. This is a structural maintenance metric, not a run-time performance metric.

**Figure 15:**
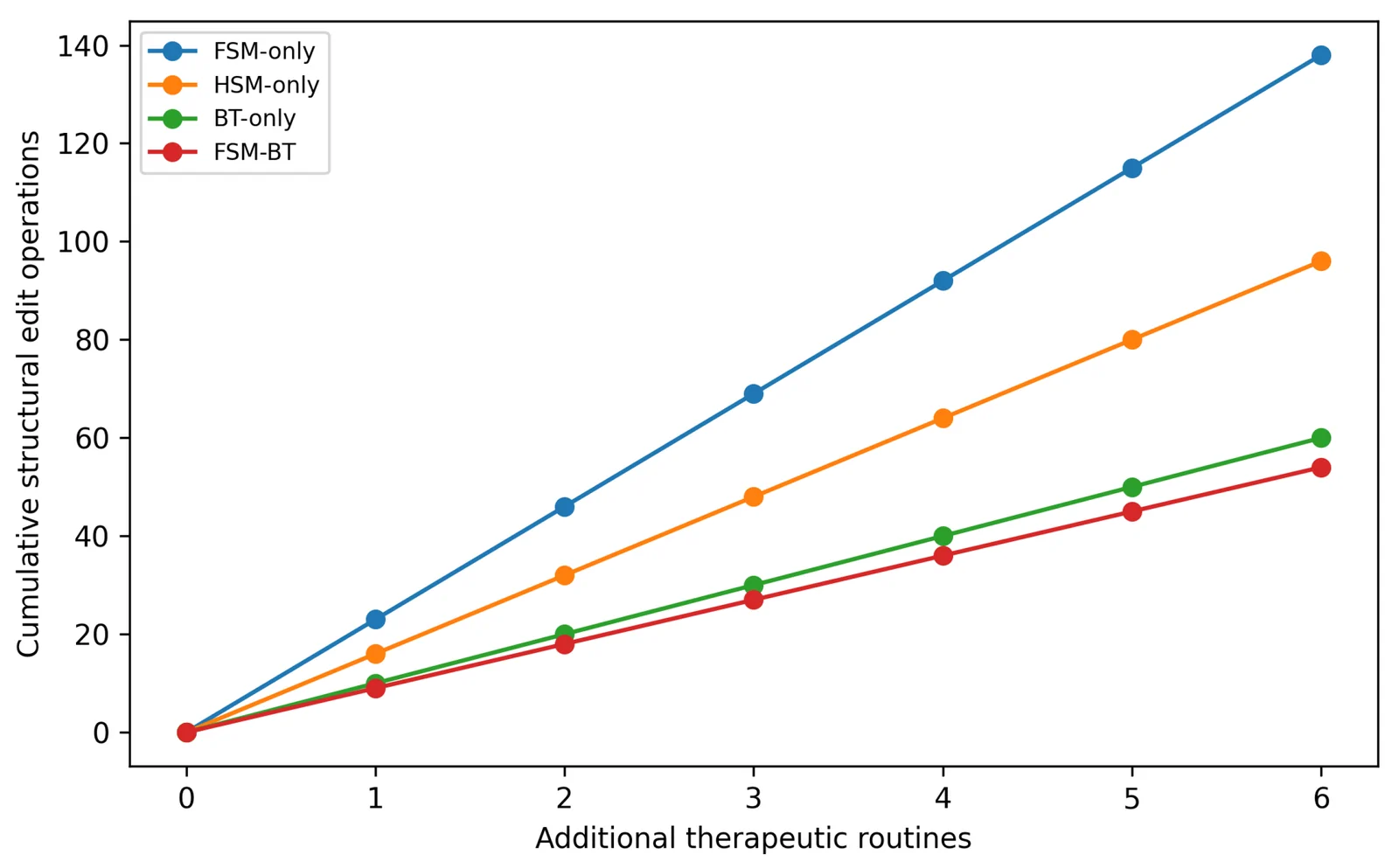
Cumulative structural edit operations required when adding up to six therapeutic routines to equivalent policy representations.

With six additional routines, the cumulative edit counts were 138 for FSM-only, 96 for HSM-only, 60 for BT-only, and 54 for FSM–BT, as illustrated in Figure 15. In the hybrid, the nine supervisory states and seventeen transitions remain unchanged because all six additions are in-mode behaviors; only BT subtrees are extended. This result provides concrete evidence for the narrower scalability claim made in this paper: *local therapeutic routine extension can be performed with less modification of the global supervisory policy*.

The result should not be generalized to every possible extension. If a new function changes the set of global operating modes or global safety semantics, the hybrid FSM must also be modified. The node-addition procedure described in Section 4 makes this boundary explicit.

### 5.11. Discussion of Results

The comparative results support a specific, bounded conclusion. The principal advantage of the proposed design is not that combining FSMs and BTs is universally superior, but that the rehabilitation-oriented allocation of responsibilities separates two different sources of complexity. Global therapeutic modes and high-priority safety escalation remain explicit in the FSM, while frequent local checks, motion-execution logic, and bounded recovery remain inside reusable BT modules. This explains why FSM–BT retained 100% S3 mode consistency while achieving the same fast 50 Hz safety detection as BT-only.

The failure analysis also explains the observed success rates. FSM-only performed worst in S2 because transient anomalies that could have been retried locally instead required global recovery logic at the slower supervisory cadence. BT-only improved local recovery, but the intentionally stressful simultaneous events in S3 exposed the absence of an explicit global-mode variable in the monolithic BT encoding. The hybrid still failed 2 of 120 trials because the injected transient exceeded both the two local retries and the single global recovery attempt; therefore, the architecture does not eliminate failure, and the results should not be interpreted as proof of fault tolerance under arbitrary disturbances.

The safety result must similarly be interpreted within scope. A 71.9 ms software response demonstrates how the 50 Hz BT and four-confirmation rule behave in the SIL scheduler. It does not include motor-drive disable time, brake dynamics, exoskeleton inertia, human reaction, or physical stopping distance. Hardware-in-the-Loop and prototype tests are required before any physical safety claim can be made.

Finally, the present plant is intentionally simplified and has no musculoskeletal human model. The shared PD controller and sinusoidal reference make the architecture comparison reproducible, but they do not represent the full nonlinear coupled dynamics of human gait or patient-specific impairment. Recent knee-exoskeleton control studies illustrate the sophistication of low-level disturbance-rejection problems in rehabilitation robotics [34,35]. The present contribution is complementary: it studies supervisory policy organization rather than proposing a new nonlinear joint controller.

### 5.12. Limitations

The study is limited to Software-in-the-Loop validation. The eight-DOF plant is a decoupled second-order model; no rigid-body multibody coupling, actuator electrical dynamics, musculoskeletal human model, therapist interaction model, or patient pathology is included. The reported latency and packet loss are stochastic inputs rather than measurements, and the safety timing ends at the software *Emergency Stop* state. The remote-control branch shown in the conceptual architecture is not quantitatively evaluated. Finally, structural edit cost is a reproducible engineering proxy for extensibility, not a universal measure of software-development effort.

## 6. Conclusions and Future Work

### 6.1. Conclusions

This work presents a rehabilitation-specific hierarchical FSM–BT supervisor–executor architecture for the SARA passive–active exoskeleton concept. The contribution is the allocation of explicit therapeutic-mode and safety authority to an FSM while parameterizable BTs execute local therapeutic behavior and bounded recovery; the generic FSM–BT pattern itself is not claimed as new.

Under the reproducible Python SIL conditions used here, the proposed architecture achieved 98.3% overall trial success, compared with 89.2% for FSM-only and 92.5% for BT-only. The hybrid software safety response averaged 71.9 ms, compared with 159.7 ms for FSM-only, while BT-only showed the same 71.9 ms response because it shared the 50 Hz local evaluation cadence. The common low-level controller produced a global eight-DOF RMSE of 2.42°. In the structural extension study, adding six local therapeutic routines required 54 edit operations for FSM–BT, 60 for BT-only, 96 for HSM-only, and 138 for FSM-only. These results support the proposed separation for the evaluated SIL task set, particularly when explicit global-mode semantics and local recovery are required simultaneously.

The conclusions are restricted to software-level architectural behavior. No claim is made regarding clinical efficacy, physical stopping time, hardware utilization, or measured STM32–ZED communication performance.

### 6.2. Future Work

Future work will proceed in stages. First, the same controller will be executed in Hardware-in-the-Loop tests with the target STM32H753ZI/ZED processing chain to measure actual scheduling, communication latency, and command-dispatch timing. Second, the complete physical SARA prototype will be used to characterize actuator inhibition, braking, mechanical emergency-stop response, and coupled multibody dynamics. Third, after engineering safety validation, experiments with healthy participants will be considered under the required institutional ethical approval and informed-consent procedures, followed by appropriately designed studies with patients and clinical outcome measures. Additional work will incorporate patient-specific assistance adaptation, a biomechanical human model, remote-monitoring validation, and comparisons with alternative supervisory architectures beyond the FSM, HSM, and BT baselines studied here.

## Figures and Tables

**Table 3:** Relevant works integrating Finite-State Machines and Behavior Trees in robotic systems.

Reference	Architecture Level	Combined Use of FSM and BT	Robotic Application
[10,14]	Hierarchical control architecture	Finite-State Machines are used to define the system’s global modes (e.g., exploration, task execution, and fault recovery), while Behavior Trees are employed within each state to organize complex actions in a modular and reactive manner. This combination preserves a clear global structure without sacrificing flexibility in behavior execution.	Autonomous mobile robots for navigation and task execution
[7,26]	High-level control and decision-making	The work proposes the use of FSMs to manage transitions between main behaviors, while Behavior Trees encapsulate the internal logic of each behavior. The FSM acts as a supervisor, and BTs enable reuse, hierarchy, and interruption handling during execution.	Mobile robotics and reactive autonomous systems
[26,27]	Task planning and execution	The Finite-State Machine handles the overall task sequence (initialization, execution, completion), while Behavior Trees control the detailed execution of actions within each state. This integration improves system robustness against unexpected events.	Service robots in dynamic environments
[8,10]	Hybrid deliberative–reactive architecture	The system uses FSMs to coordinate high-level deliberative behaviors, such as task selection, and Behavior Trees for reactive and hierarchical execution of those tasks. BTs enable rapid responses to environmental changes without altering the FSM’s global structure.	Social and assistive robots
[9,28]	Hierarchical behavior control	The FSM defines the robot’s global operational states, while Behavior Trees organize subtasks and actions within each state. This combination facilitates debugging and system extension without requiring a complete redesign of the architecture.	Humanoid robots and autonomous platforms

**Table 4:** Behavior and control models employed in robotic exoskeletons.

Behavior Model	Technical Description	Exoskeleton Examples and References
Finite-State Machines (FSMs)	Discrete model based on a finite set of states and transitions, widely used to segment human gait into phases (stance, swing, double support) and to manage operating modes such as rest, active assistance, and emergency stop. It offers high interpretability and ease of verification, which are critical aspects in rehabilitation systems.	BLEEX exoskeleton and its derivatives for gait assistance [29]; ReWalk exoskeleton for lower-limb rehabilitation [30]; clinical exoskeletons reviewed by Dollar and Herr [1].
Hierarchical State Machines (HSMs)	Extension of FSMs that incorporates hierarchy and substates, allowing complex behaviors to be organized through multiple levels of abstraction. It is used to reduce state explosion in complex therapies and to structure assistance modes with different levels of detail.	Hierarchical control systems in powered exoskeletons for assisted walking [31]; hierarchical architectures in human augmentation exoskeletons [29].
Behavior Trees (BTs)	Hierarchical and modular model based on control, condition, and action nodes, enabling reactive behavior execution. It facilitates the integration of assistance actions, user monitoring, and safety supervision, allowing continuous reevaluation of system conditions.	Emerging applications in rehabilitation exoskeletons with modular and reactive control [6]; assistive robotic systems with BT-based architectures adapted to human–robot interaction [22].
Rule-Based Systems	Logical model based on if–then rules, used to adjust assistance parameters such as torque, stiffness, or control gains according to biomechanical or clinical variables. It stands out for its interpretability and ease of tuning by clinical personnel.	Post-stroke rehabilitation exoskeletons with performance-based assistance adjustment [2]; assistive systems reviewed in [1].
Probabilistic Models (MDP, POMDP)	Decision-making models under uncertainty that enable user intention inference and action selection by considering transition probabilities and rewards. They are particularly useful when the user’s state is not fully observable.	Exoskeletons and assistive devices with intention recognition based on MDPs; adaptive assistance systems under uncertainty [32].
Reinforcement Learning (RL)	Learning-based approach that enables the system to optimize assistance policies through interaction with the user. It is used to personalize rehabilitation and progressively adapt assistance levels, typically integrated within supervised control architectures.	User-centered adaptive control in rehabilitation exoskeletons [18]; recent control trends in rehabilitation exoskeletons [33].

**Table 5:** Pseudocode for the three control-policy representations used in this study.

Policy	Pseudocode
FSM-only	1. Read events and safety guards. 2. Evaluate outgoing transitions from the current state according to priority. 3. If a valid guard is satisfied, update the global state. 4. Execute the action associated with that state. 5. Represent transient recovery as explicit recovery states/transitions. 6. Repeat at 10 Hz.
BT-only	1. Tick the root at 50 Hz. 2. Evaluate global-mode selection and local safety branches within the same tree. 3. Traverse condition and action nodes. 4. Execute local recovery for at most $R_{BT}$ attempts. 5. Propagate *Success*, *Failure*, or *Running* to the root. 6. Re-tick the tree.
FSM–BT	1. FSM selects and authorizes a global therapeutic mode at 10 Hz. 2. Activate Γ(s). 3. Tick the associated BT at 50 Hz. 4. Execute local condition/action nodes and bounded recovery. 5. If the BT succeeds, report completion; if local recovery fails, emit $e_{esc}$; if a critical guard is detected, emit a high-priority safety event. 6. FSM processes completion/escalation and updates the global state.

**Table 6:** SARA sensing configuration represented in the SIL model.

Sensor Group	Count	SIL Role
Integrated torque sensors	8	Joint effort and unexpected-resistance supervision.
Absolute encoders	8	Joint position feedback for tracking and joint-limit checks.
MPU6050 IMUs	3	Pelvis/thigh orientation and user-stability checks.
FSR-402 plantar sensors	24	Normalized plantar-load distribution and support asymmetry.
OP5315W2 optical limit sensors	16	Two virtual limit channels per actuator for joint-limit proximity.
Total	59	Software-represented sensing elements; no physical acquisition in this study.

**Table 7:** Eight-coordinate SIL plant, reference, and low-level controller parameters. Values are simplified equivalent simulation parameters and are not identified physical SARA parameters.

Coordinates	$I_{i}$	$b_{i}$	$A_{i}$ (Deg)	$f_{i}$ (Hz)	$K_{p,i}$	$K_{d,i}$
Hip 1–2	1.60	1.20	18	0.25	22	4.0
Hip 3–4	1.20	1.00	12	0.22	18	3.5
Knee 1–2	0.80	0.80	28	0.22	24	4.5
Ankle 1–2	0.60	0.60	8	0.30	16	3.0

**Table 8:** Main event categories used by the implemented FSM.

Event Category	Representative Implementation Examples
Sensory events	Joint angle validation, interaction force or torque threshold, load distribution, posture condition, inertial orientation.
Interface events	Therapist start command, stop command, mode selection, parameter update, routine confirmation.
System events	Calibration result, communication status, actuator availability, battery condition, BT execution status.
Safety events	Emergency stop, joint-limit violation, user instability, excessive force, persistent execution fault.

**Table 9:** BT node counts used in the SARA SIL implementation.

Behavior Tree	Control	Condition	Action	Recovery	Total
System Initialization	3	4	4	4	15
Therapy Configuration	3	5	5	5	18
Exercise Execution	3	5	5	5	18
Total	9	14	14	14	51

**Table 10:** Recovery logic and escalation used by the proposed architecture.

Condition	Local BT Response	Persistent/Critical Response
Transient communication anomaly	Retry operation and revalidate link	FSM Recovery; timeout/three consecutive losses → emergency stop/fault
Sensor inconsistency	Re-read/revalidate sensor set	FSM recovery if inconsistency persists
Posture/load instability	Suspend action and revalidate stability	Recovery; critical instability → emergency stop
Unexpected resistance	Reduce/hold assistance and reevaluate	Emergency stop if threshold remains exceeded
Joint-limit proximity	Inhibit motion toward the limit	Immediate emergency stop if the unsafe guard persists

**Table 11:** Main SIL conditions used for all architectural comparisons.

Parameter	Value
Plant integration	100 Hz, Δt=0.01 s
FSM update	10 Hz, $T_{FSM}=100$ ms
BT tick	50 Hz, $T_{BT}=20$ ms
Trial duration	8.0 s
Initial conditions	$q_{i}\left(0\right)=0$, ${\dot{q}}_{i}\left(0\right)=0$ for all eight coordinates
Monte Carlo trials	30/scenario/architecture; 360 total
Random seed	2026
Communication latency	$L=\max(0,L_{s})$, $L_{s}∼N\left(9,{\left(2\right)}^{2}\right)$ ms
Packet loss	Bernoulli process, $p_{loss}=0.008$
Encoder noise	Gaussian, $\sigma_{q}=0.25{}^{\circ}$
Interaction-torque noise	Gaussian, $\sigma_{\tau }=0.30$ Nm
Safety confirmation	Four consecutive unsafe BT evaluations
Safety thresholds	5° joint margin; 12 Nm resistance; 5° sensor inconsistency; 10° instability; 50 ms communication timeout

**Table 12:** Functional validation scenarios.

Scenario	Description	Primary Objective
S1: Passive Mode	User-driven/passive operation with virtual sensing and safety supervision.	Verify safe supervision without active reference tracking.
S2: Active Assistance	Eight-DOF cyclic reference tracking with stochastic sensing/interaction noise and recoverable transient events.	Evaluate tracking and local recovery.
S3: Passive–Active Transition	Valid mode request, BT reactivation, and occasional simultaneous completion/request events.	Evaluate FSM–BT coordination and mode consistency.
S4: Safety Interruption	One simulated unsafe condition is forced beyond its configured threshold.	Evaluate software detection, escalation, and stop-state entry.

**Table 13:** Active-assistance tracking indicators for the common low-level SIL controller.

Indicator	Result
Global eight-DOF RMSE	2.424±0.008°
Global eight-DOF MAE	1.764±0.009°
NRMSE (joint range normalization)	7.49±0.04%
Mean peak commanded torque	9.20±0.08 Nm

**Table 14:** Architecture-level SIL comparison under matched Monte Carlo conditions.

Architecture	Overall Success	Recovery Success	S3 Mode Consistency	Safety Response
	% (95% CI)	%	%	ms, Mean ± SD
FSM-only	89.2 (82.3–93.6)	59.4	100.0	159.7±26.3
BT-only	92.5 (86.4–96.0)	81.3	90.0	71.9±5.3
FSM–BT	**98.3 (94.1–99.5)**	**93.8**	**100.0**	71.9±5.3

## Data Availability

The revised submission ZIP includes the Python SIL source code, dependency list, generated trial-level CSV files, summary statistics, and generated comparison figures used to reproduce the reported numerical results.
